# Alfalfa-Yolo: a boundary-aware lightweight framework for real-time weed detection in alfalfa fields

**DOI:** 10.3389/fpls.2026.1955970

**Published:** 2026-10-15

**Authors:** Jingyi Wang, Kai Li, Tao Zhou, Yuanjun Xu, Yiwen Gao, Changzhong Li, Yanhua Zhao

**Affiliations:** 1 College of Grassland Science, Qingdao Agricultural University, Qingdao, Shandong, China; 2 Shandong Key Laboratory for Germplasm Innovation of Saline-alkaline Tolerant Grasses and Trees, Qingdao, Shandong, China

**Keywords:** alfalfa, edge computing, feature pyramid, precision agriculture, weed detection, YOLO

## Abstract

Accurate weed detection in alfalfa fields is hindered by morphological similarity between crops and weeds at the seedling stage, substantial scale variation across weed targets, and degradation of fine spatial information during feature resampling. To address these challenges, Alfalfa-Yolo is proposed as a boundary-aware compact detection framework integrating three complementary components. The Edge-Perception Global Transfer Network (EPGTNet) uses bidirectional Sobel responses and cross-layer transmission to preserve edge-related high-frequency cues in deeper semantic features. The Multi-Scale Adaptive Feature Pyramid Network (MSAFPN) replaces static concatenation with attention-guided aggregation to adaptively regulate multi-scale contributions. The Enhanced Channel Shuffle Upsampling Module (ECSM) combines depthwise convolution, channel shuffle, and spatial shift to enhance local spatial representation after upsampling. On the self-constructed alfalfa-field dataset comprising 1,100 independent original field images, Alfalfa-Yolo achieves 77.6% mAP@0.5 and 55.9% mAP@0.5:0.95 with 2.67 M parameters. Independent-dataset benchmarking on CottonWeedDet12 and ImageWeeds confirms that the architecture remains competitive after target-dataset retraining, while a separate zero-shot cross-dataset protocol directly evaluates transfer without target-domain training. Deployment tests on NVIDIA RTX 4090, NVIDIA Orin NX, and RK3588 further characterize the accuracy-efficiency trade-off under different hardware and quantization settings. These results indicate that Alfalfa-Yolo provides a compact and practically deployable solution for real-time weed localization in alfalfa fields.

## 1 Introduction

Alfalfa (*Medicago sativa*), known as the “Queen of Forages”, is one of the world’s most important leguminous forage crops, cultivated on more than 30 million hectares worldwide (Zhang and Wang, 2025). It is highly nutritious and used in the livestock industry as an excellent feed and an important forage crop. However, weeding is associated with being one of the major limiting factors that reduce alfalfa yield. Weeds compete with alfalfa for light, water and nutrients, reducing yield by over 34% on average in badly infected fields (Moyer et al., 1991). Herbicide sprayings of broad-spectrum chemicals lead to environmental pollution and food safety problems, while also herding resistant weed populations. Manual weeding is labor-intensive, costly and inefficient. Therefore, research about precise, robust, economical and environmentally friendly weed detection and management technologies applicable to the alfalfa field are important to allow sustainable agricultural development, improve alfalfa yield, and protect ecological environments. Classical methods of weed detection are mainly based on machine vision techniques which extract shallow features like texture, shape, color or other spectral characteristics and feed them to classifiers for the task of recognition (Wu et al., 2021). Early weed detection systems used color difference based methods which exploited the differences in the spectral reflectance of green plants and the soil backgrounds, or used interrow detection methods which classify any plant growing between a crop row as a weed (Wang et al., 2019). While the traditional approaches can indeed allow for weed detection under specific conditions, they are often encumbered with problems of feature extraction, poor environmental adaptability, or if any are available, an accuracy of detection.

In recent years, deep learning techniques (Liakos et al., 2018) have made remarkable advances in agricultural applications, offering new solutions for weed detection. Compared with conventional machine learning methods, deep learning algorithms can automatically learn high-level abstract features, significantly improving the accuracy and robustness of weed recognition (Murad et al., 2023). Yang et al. (2022) compared the performance of Faster R-CNN, VGGNet, and YOLOv3 for detecting weeds growing in alfalfa fields, finding that VGGNet achieved the best performance for broadleaf and grass weed detection, with F1 scores exceeding 0.99. But these results do not necessarily represent real-time object detection performance in dense, seedling-stage field scenes or on resource-constrained edge devices. With the continued development of deep learning, object detection algorithms have achieved significant progress in both detection accuracy and real-time performance, laying a solid technical foundation for precise and real-time weed detection (Olsen et al., 2019).

The algorithms of object detection have shown great promise in the weed detection arena, and the two broad classes of algorithms are detected by two-stage and one-stage algorithms. Veeranampalayam Sivakumar et al. created Faster R-CNN and SSD models for soybean field weed detection in UAV imagery and discovered that Faster R-CNN improved detection performance over SSD (Veeranampalayam Sivakumar et al., 2020). Although two-stage algorithms achieve outstanding performances in detection, the high computation complexity makes it challenging to employ them for real-time. In comparison to it, single-stage object detection algorithms not only guarantee the detection accuracy, but also greatly enhance the detection speed, making real agricultural applications viable.

As the YOLO (Redmon et al., 2016) series have gone through iterations one by one, researchers have proposed various improvement schemes to enhance weed detection performance. Chen et al. proposed YOLO-sesame, an improved YOLOv4 with enhanced attention mechanisms and feature fusion to effectively boost the weed detection performance in sesame fields (Chen et al., 2022). Wang et al. proposed the YOLO-Weed Nano algorithm based on an improved YOLOv8n, replacing the original backbone with a depthwise separable convolution-based DS_HGNetV2 network to achieve a high accuracy (Wang et al., 2025). Jia et al. proposed ADLYOLOv8, which incorporates a lightweight AKConv block and an ultra-lightweight DySample upsampling module, reducing model complexity while improving detection accuracy by 2.2% (Jia et al., 2024). Ma et al. proposed YOLOCWD, achieving a lightweight design through a hybrid attention mechanism and IoU loss function, for corn field weed detection achieving a mAP@50 of 75.1% with only 9.6 GFLOPs (Ma et al., 2025). Sharma et al. conducted a thorough performance comparison of YOLOv8, YOLOv9, YOLOv10, YOLOv11, and Faster R-CNN on multiple weed detection tasks, concluding that YOLOv11 achieved the fastest inference speed, while YOLOv9 achieved the highest detection accuracy (Sharma et al., 2024). Sun et al. proposed the AGRI-YOLO lightweight corn field weed detection model based on the YOLO v11n architecture, reconstructing the C3k2 feature extraction module with InceptionNeXt DWConv and replacing the Conv layers with ADown adaptive downsampling to cut down redundant parameters (Peng et al., 2025). Yang et al. proposed GTDR-YOLOv12, the goal of which is real-time weed recognition in field-scale complex outdoor environments, they improved the accuracy from 85.0% to 88.0%, and GFLOPs from 5.8 to 4.8 compared to YOLOv12 (Yang et al., 2025).These studies demonstrate that introducing attention mechanisms, optimizing multi-scale feature fusion, and improving small-target detection capability are effective strategies for enhancing YOLO-based weed detection performance.

Recent vision-language models provide a complementary direction for agricultural perception through text-guided and zero-shot recognition. Nasir et al. (2026) evaluated modern vision-language models for zero-shot weed presence detection, spatial localization, crop growth-stage recognition, and visual reasoning using UAV imagery. Deng and Lu (2026) further investigated Grounding DINO, YOLO-World, and Qwen3-VL for crop-weed detection and cross-season generalization, illustrating the potential of language-guided perception under changing data distributions.

These approaches address a different operating regime from the detector developed in this study. Vision-language models are attractive when target categories change frequently, annotation is limited, or semantic reasoning is required. In contrast, Alfalfa-Yolo targets a predefined closed-set field task requiring repeated low-latency inference and deployment on compact computing platforms. Under fixed crop and weed categories and constrained edge-computing budgets, a compact fully supervised detector remains advantageous in computational cost, deterministic output format, and deployment simplicity. Vision-language models are therefore regarded as a complementary direction for future open-world agricultural perception rather than a direct replacement for the present closed-set detector.

Despite the advances that detector based on YOLO have achieved in weed detection, there are still practical challenges to be solved. (1) Alfalfa and weeds are also very similar in morphology in the seedling stage, with little difference in color and shape. And traditional simple fixed-receptive field convolutions still cannot adaptively fit the weed target of varying size in multiple scales, thus limiting the detection accuracy. (2) Each form of downsampling is based on a fixed spatial-domain sampling method, which will inevitably lose part of the high-frequency detail information in the process of dimensionality reduction, resulting in a serious loss of performance for small weed targets. (3) Traditional attention mechanisms rely excessively on single global self-attention and lack fine-grained modeling of inter-channel semantic dependencies and spatial position information, failing to fully exploit the complementary information across feature channels.

To address the key limitations of conventional YOLOv11 in weed recognition tasks, including attenuation of edge-related detail, insufficient adaptive multi-scale fusion, and spatial-detail loss during upsampling, Alfalfa-Yolo is proposed. The main contributions are summarized as follows:

- Edge-Perception Global Transfer Network (EPGTNet). An end-to-end edge-information transmission architecture is constructed to preserve and propagate boundary-related high-frequency cues that are easily attenuated in the standard YOLOv11 backbone. The network contains the Adaptive Multi-Scale Edge Feature Generator (AMSEFG) and Progressive Edge-Semantic Fusion Module (PESFM), which combine bidirectional Sobel responses with cross-layer transmission and semantic fusion.
- Multi-Scale Adaptive Feature Pyramid Network (MSAFPN). A multi-level bidirectional feature-fusion architecture is constructed to overcome the fixed contribution of simple concatenation. Guided by the Attention-Guided Multi-Feature Aggregation Module (AMFAM), MSAFPN performs adaptive weighted fusion of multi-scale features to improve representation of weed targets with different sizes and visual contexts.
- Enhanced Channel Shuffle Upsampling Module (ECSM). A lightweight hierarchical feature-enhancement module combines depthwise convolution, channel shuffle, and the Spatial-Temporal Channel Redistribution Unit (STCRU) to enhance local spatial representation after upsampling. The design targets fine-detail attenuation and block-like local transitions associated with fixed interpolation rather than treating checkerboard artifacts as an inherent property of nearest-neighbor interpolation.

## 2 Related work and baseline

### 2.1 Self-constructed dataset

The dataset used in this study was constructed through field surveys at the Modern Demonstration Park of Qingdao Agricultural University in Jiaozhou, Qingdao, Shandong Province, China. Image collection was conducted between April and September 2025. A total of 3,589 raw images were initially acquired, of which 1,100 high-quality original field images were retained after quality screening. Seven common weed species were represented: Chenopodium album, Chenopodium glaucum, Amaranthus retroflexus, Portulaca oleracea, Abutilon theophrasti, Calystegia hederacea, and Cyperus rotundus. Representative labeled images of weeds and alfalfa from the self-constructed dataset are shown in **Figure 1**. Although multiple weed species are present, all weed instances are intentionally mapped to a single generic weed category for model training. The intended task is weed localization for subsequent site-specific control during the alfalfa seedling stage rather than species-level weed recognition. This label design is compatible with mechanical or generic spot-treatment applications, but it does not support species-specific management decisions or evaluate discrimination among morphologically similar weed species. All images were collected during the alfalfa seedling management period.

**Figure 1:**
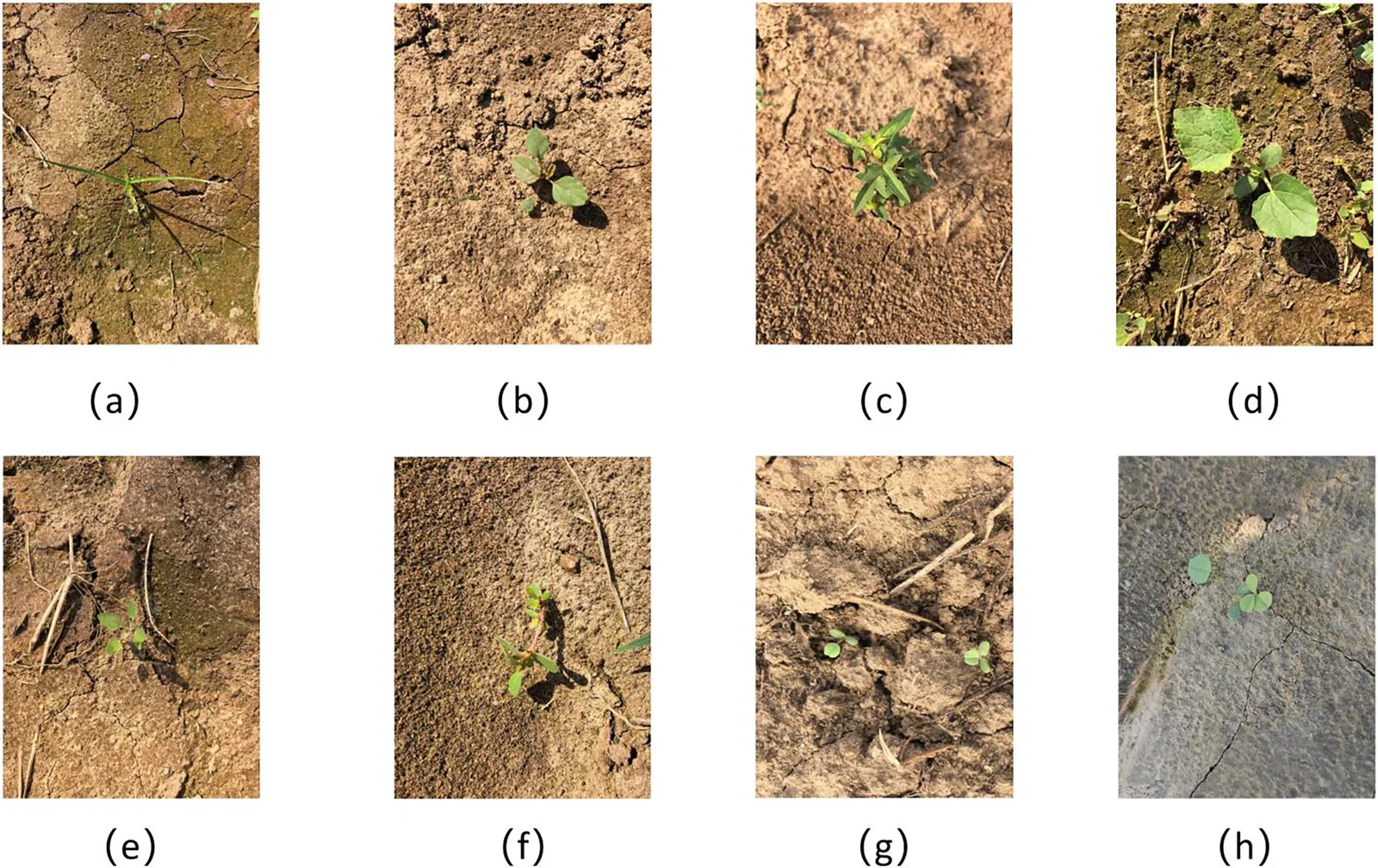
Representative labeled images of the seven weed species in the self-constructed dataset. **(A)** *Cyperus rotundus*; **(B)** *Amaranthus retroflexus*; **(C)** *Calystegia hederacea*; **(D)** *Abutilon theophrasti*; **(E)** *Chenopodium album*; **(F)** *Portulaca oleracea*; **(G, H)** *Medicago sativa*.

The original field images were manually annotated using LabelImg. Bounding boxes were drawn to tightly enclose the visible extent of each target. For partially overlapping plants, individual instances were annotated separately when their visible structures allowed reliable instance separation. Severely occluded or visually ambiguous regions for which an individual target boundary could not be determined reliably were not forced into uncertain annotations.

The first annotation pass was subsequently checked through a manual cross-check procedure. Quality control focused on missing objects, incorrect category assignments, and excessively loose or truncated bounding boxes. Disagreements were resolved by re-examining the original image before the labels were used for training. The manual cross-check was used as the annotation quality-control procedure rather than a separate statistical inter-annotator agreement analysis.

To reduce correlation between the training and evaluation data caused by consecutive field acquisition, dataset partitioning was performed at the acquisition-group level rather than by unrestricted random image-level splitting. Original images were organized according to field location, acquisition session, and continuous capture sequence. Images from the same location within the same continuous acquisition sequence were treated as a single group and assigned collectively to only one of the training, validation, or test subsets. Consequently, no sequence from the same acquisition group occurred simultaneously in the training and test sets. Partitioning was completed before subsequent data augmentation, and all model comparisons used this fixed group-wise split.

**Table 1** summarizes the independent original observations used for model development and evaluation. The training, validation, and test subsets contain 880, 110, and 110 original images, respectively, with 5,190, 646, and 650 annotated weed instances. Offline augmentation is restricted to the training subset; validation and test images remain unaugmented original field observations. This protocol prevents augmented derivatives or closely related acquisition sequences from crossing subset boundaries.

**Table 1:** Partition statistics of the original self-constructed field dataset.

Subset	Original images	Proportion	Weed instances	Offline augmentation
Training	880	80%	5190	Training only
Validation	110	10%	646	Not used
Test	110	10%	650	Not used
Total	1100	100%	6486	Training subset only

To characterize dataset composition more transparently, weed-instance statistics were further summarized by species, target size, environmental condition, and data subset using only the original field observations. Offline augmented images are excluded from these counts because they do not constitute additional independent field observations. Species-level statistics describe the dataset composition, whereas the detector itself retains the single generic weed label. Target-size categories are determined from bounding-box area under the unified input scale. Because fine-grained weed phenological substages were not independently recorded during acquisition, no retrospective substage labels were introduced beyond the documented alfalfa seedling management period.

As shown in **Table 2**, all seven weed species occur in each subset, and the dominant target population is composed of small and medium objects, which together account for 5,855 of the 6,486 annotated weed instances. Sunny, overcast, and post-rain conditions are also represented across training, validation, and test data. The distribution therefore preserves the principal species, scale, and environmental variations needed for controlled evaluation while maintaining acquisition-group independence.

**Table 2:** Distribution of weed instances by species, target size, environmental condition, and data subset.

Data attribute	Training	Validation	Test	Total
*Chenopodium album*	963	120	121	1204
*Chenopodium glaucum*	699	86	87	872
*Amaranthus retroflexus*	868	108	110	1086
*Portulaca oleracea*	753	95	96	944
*Abutilon theophrasti*	549	68	67	684
*Calystegia hederacea*	588	74	74	736
*Cyperus rotundus*	770	95	95	960
Small targets	2824	350	350	3524
Medium targets	1862	232	237	2331
Large targets	504	64	63	631
Sunny condition	2280	285	285	2850
Overcast condition	1710	213	217	2140
Post-rain condition	1200	148	148	1496
Total weed instances	5190	646	650	6486

To enhance model robustness, field images were collected under different weather conditions and vegetation-density patterns. As illustrated in **Figure 2**, the density descriptors indicate relative differences among the displayed field examples rather than defining an extreme occlusion benchmark. Higher-density conditions contain more closely spaced weed instances and greater local overlap or partial occlusion than the sparse condition. After the group-wise split was fixed, horizontal flipping, brightness adjustment, rotation at multiple angles, noise addition, and blur processing (Shorten and Khoshgoftaar, 2019) were applied only to the training subset. **Figure 3** illustrates these augmentation operations; validation and test observations were not augmented.

**Figure 2:**
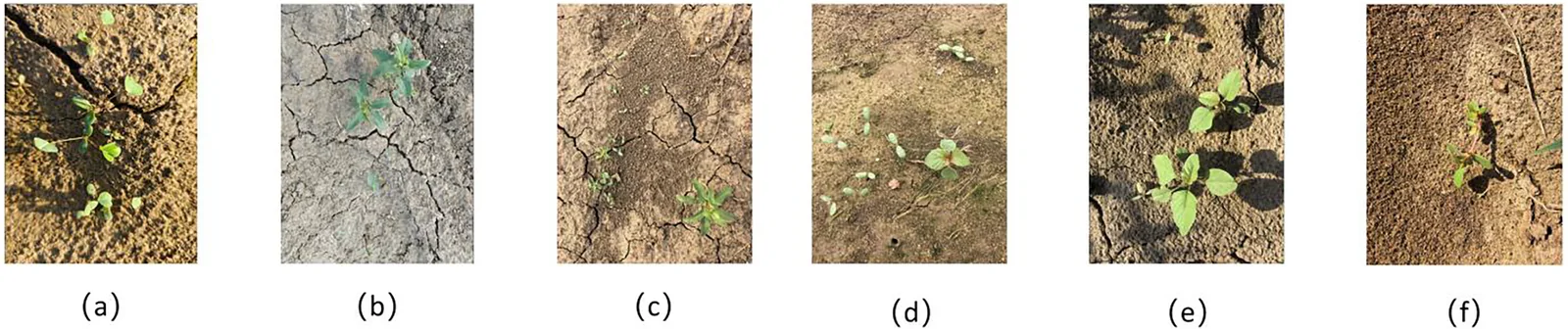
Representative images from the self-constructed dataset under diverse environmental conditions. **(A)** Sunny weather; **(B)** overcast weather; **(C)** post-rain conditions; **(D)** relatively high-density weed condition; **(E)** moderately dense weed condition; **(F)** sparse weed condition.

**Figure 3:**
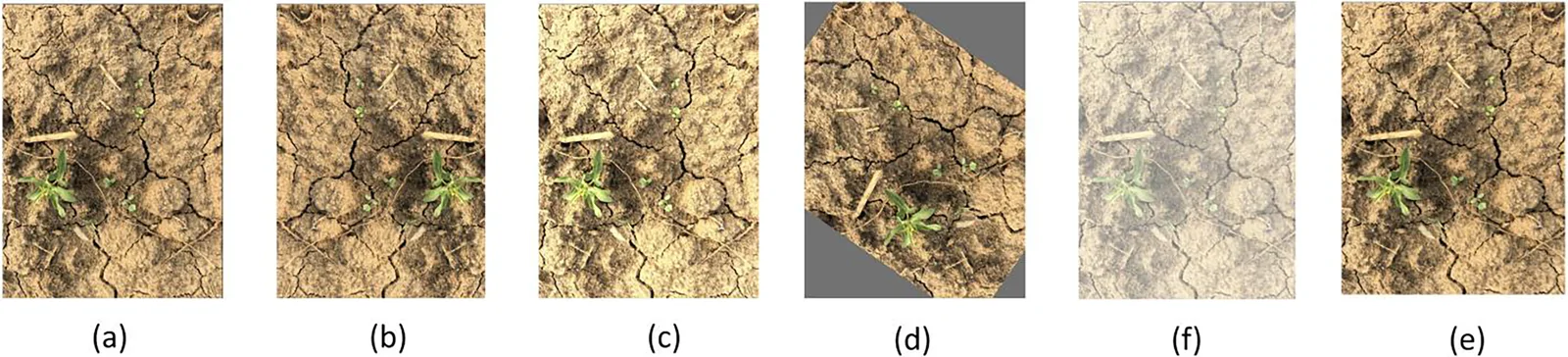
Illustration of data augmentation strategies. **(A)** Original image; **(B)** horizontal flip; **(C)** brightness adjustment; **(D)** rotation; **(E)** blur processing; **(F)** noise addition.

### 2.2 YOLOv11 baseline model

YOLOv11 (Khanam and Hussain, 2024), published by Ultralytics in 2024, is the latest version in the series and represents the state of the art in the field in terms of accuracy and speed. YOLOv11 implements a new architecture, including an enhanced backbone, improved feature fusion, and new, improved head structures, while remaining real-time, with a larger improvement in performance.

The backbone uses a revised Cross Stage Partial (CSP) architecture, with the new C3k2 module being a lightweight feature extraction module with a good trade-off between computational budget and feature representation capabilities. These are then gradually extracted in the network backbone as five feature maps from P1/2 to P5/32 through several downsample layers running multi-scale feature extraction. Also relieving the workload on the backbone for a faster process, the embedded Spatial Pyramid Pooling-Fast (SPPF) module efficiently makes the receptive field bigger by pooling across scales, significantly cutting the computational complexity of the standard SPP module while providing the same comparable functionality.

The neck is built from the Path Aggregation Network (PAN) structure. Effective integration of high-level semantic information and low-level details is achieved via top-down and bottom-up feature fusion paths. The neck further incorporates the C2PSA (Cross-Stage Partial with Spatial Attention) module, which increases the discriminability of feature representations through the use of spatial attention. Three pyramids (P3, P4 and P5) of features at different scales are constructed by applying upsampling and downsampling operations, and the final detection of the large medium and small targets can be achieved at the same time.

YOLOv11 also utilizes a decoupled detection head, where the classification and regression predictions are made from separate branches, which speeds up convergence and improves detection performance. The head contains 3 output layers for the P3, P4, and P5 feature maps. Instead of anchor-based detection, this model also regresses the center point and the width and height of the targets in an end-to-end, anchor-free manner, simplifying the model and removing the need to optimize anchor hyperparameters.

## 3 Methodology

An input image is processed through a convolutional layer and two stacked C3k2 blocks to produce P2-scale feature maps, which serve as high-resolution spatial priors and are reused at multiple stages. The Adaptive Multi-Scale Edge Feature Generator (AMSEFG) takes the P2 features and produces edge-feature tensors at P3, P4, and P5 scales in parallel. Each scale-specific edge feature is fused with the corresponding semantic feature through PESFM, explicitly injecting edge-related high-frequency cues into deeper representations. The P5 features are subsequently enhanced through SPPF and C2PSA for high-level semantic representation. In the neck, a bidirectional feature pyramid based on AMFAM performs adaptive cross-scale fusion. The top-down pathway integrates backbone features, high-resolution spatial priors, and adjacent semantic complements, whereas the bottom-up pathway uses ECSM connections to redistribute spatial and semantic information among detection scales. The final P3, P4, and P5 features are fed into decoupled detection heads with strides of 8, 16, and 32, respectively. The overall architecture is shown in **Figure 4**.

**Figure 4:**
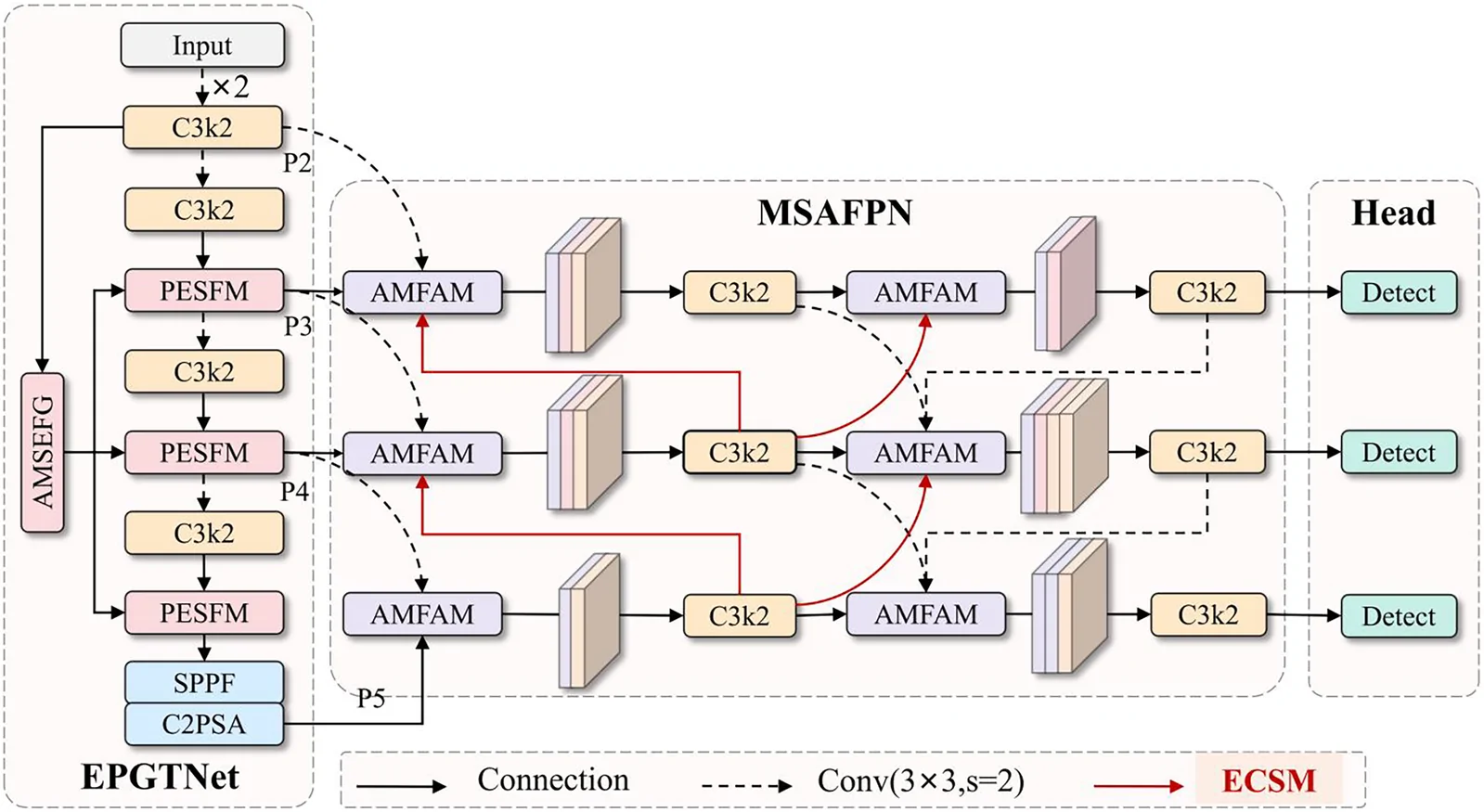
Overall architecture of the Alfalfa-Yolo algorithm.

### 3.1 Edge-perception global transfer network

Although the standard YOLOv11 backbone is effective for weed detection, repeated down-sampling can attenuate high-frequency spatial cues that are useful for distinguishing morphologically similar crop and weed regions. EPGTNet therefore constructs a global transmission pathway for edge-related information from shallow to deeper feature levels. The term boundary-aware in this study refers to the explicit use and propagation of gradient-derived high-frequency cues within a bounding-box detector; it does not imply pixel-level contour prediction. The structure of EPGTNet is illustrated in **Figure 5**.

**Figure 5:**
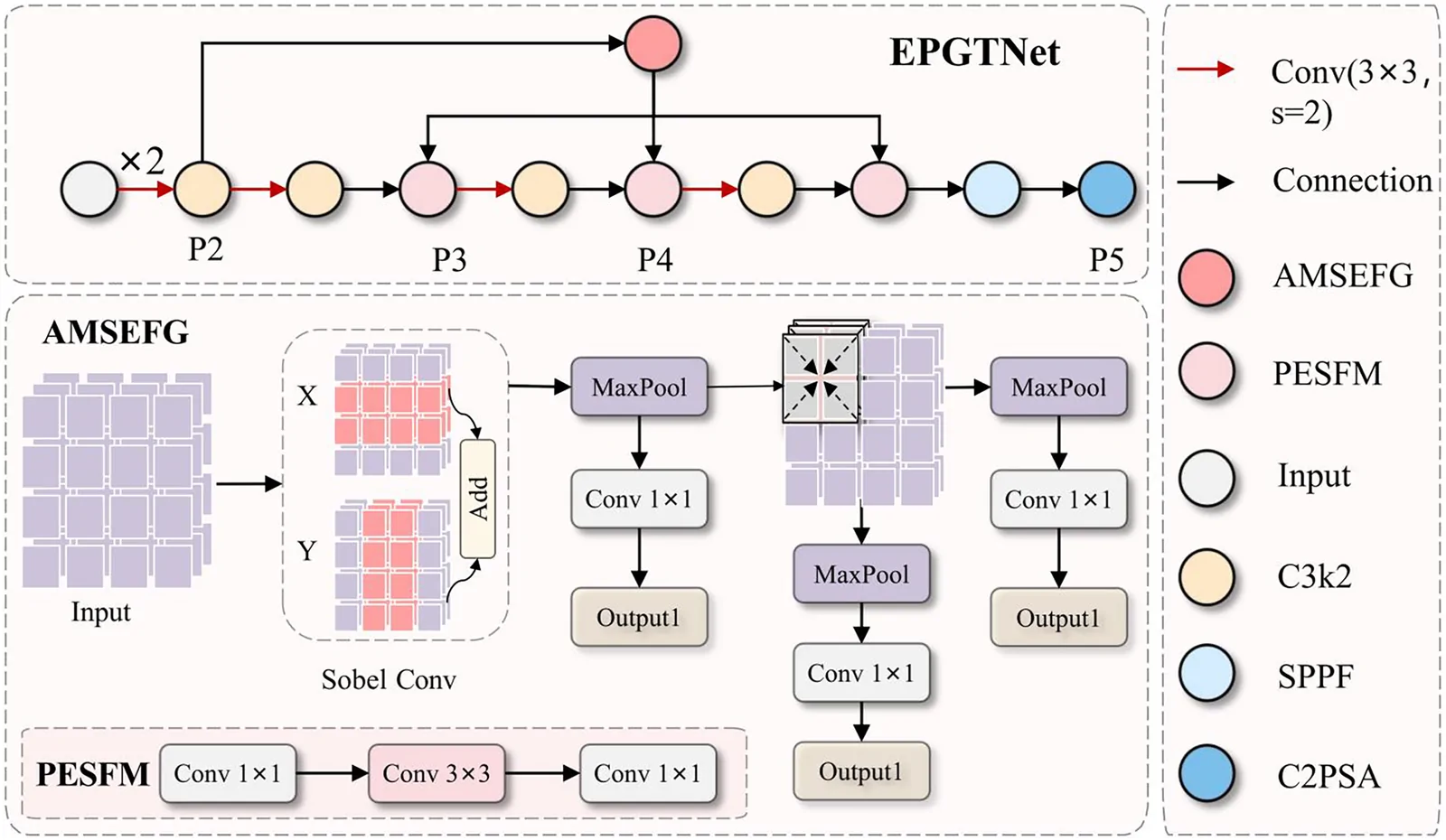
Architecture of the edge-perception global transfer network (EPGTNet).

EPGTNet adopts a three-stage perception–transmission–fusion design philosophy, constructing an end-to-end edge information global transmission architecture. At the shallow feature extraction stage, the Adaptive Multi-Scale Edge Feature Generator (AMSEFG) is introduced to generate edge feature maps at different resolutions using an improved Sobel edge detection operator. Edge information is then directly transmitted to corresponding deep feature layers via a cross-layer transmission mechanism. Finally, the Progressive Edge-Semantic Fusion Module (PESFM) organically integrates edge features with semantic features at each target level. The feature transmission process of the entire network can be formulated as shown in Equation 1:

(1) $${ℱ}_{o}^{l}={\text{PESFM}}^{l}\left({ℱ}_{s}^{l},\text{Transfer}\left(\epsilon_{s}^{0}\right)\right)$$

where $\epsilon_{s}^{0}$ denotes the edge features generated at the shallow layer, Transfer(·) represents the cross-layer transmission function, PESFM*^l^* denotes the fusion operation at the l-th level, and ${ℱ}_{s}^{l}$ and ${ℱ}_{o}^{l}$ are the convolutional feature and the fused output feature at the *l*-th level, respectively.

AMSEFG, the core module for edge information extraction, employs an improved Sobel operator for multi-scale edge detection. The module first uses 3D convolution-implemented Sobel kernels to extract horizontal and vertical gradient information. The horizontal and vertical gradient kernels are defined as shown in Equation 2:

(2) $$K_{x}=\left[\begin{array}{ccc} 1 & 0 & -1\\ 2 & 0 & -2\\ 1 & 0 & -1 \end{array}\right],\;K_{y}=\left[\begin{array}{ccc} 1 & 2 & 1\\ 0 & 0 & 0\\ -1 & -2 & -1 \end{array}\right]$$

By convolving the input feature map with these two gradient kernels, the edge intensity response is obtained as shown in Equation 3:

(3) $$\epsilon \left(i,j\right)=\sqrt{{\left(K_{x}*ℐ\left(i,j\right)\right)}^{2}+{\left(K_{y}*ℐ\left(i,j\right)\right)}^{2}}$$

where ℐ(i,j) denotes the input feature map and ∗ represents the convolution operation. The module then generates three edge feature maps at different scales through successive max-pooling operations and uses 1×1 convolutions for adaptive channel dimension adjustment to ensure that the edge features match the target level’s feature dimensions. The output edge features are transmitted to the P3/8, P4/16, and P5/32 feature levels, respectively.

PESFM achieves deep fusion of edge features and convolutional features via a progressive feature fusion strategy. The module first concatenates the transmitted edge features with the current layer’s convolutional features along the channel dimension for preliminary fusion, then applies 1×1 convolution for channel compression to reduce feature dimensionality while preserving critical information. Subsequently, 3×3 convolution is employed for spatial feature extraction to enhance the spatial representation capability of the fused features. A final 1×1 convolution restores the features to the target channel count. This fusion strategy ensures the effective incorporation of edge information, avoids feature dimensionality explosion, and achieves an optimal balance between edge features and semantic features, significantly improving the network’s discriminability of weed boundary information.

EPGTNet complements the standard YOLOv11 backbone by preserving and transmitting edge-related information across multiple feature scales. The combination of bidirectional Sobel responses and progressive edge-semantic fusion strengthens the representation of subtle spatial differences between crop and weed regions while retaining a compact backbone structure. Because the detector outputs bounding boxes rather than segmentation masks, these improvements are interpreted as enhanced use of boundary-related cues for recognition and box localization rather than explicit recovery of pixel-level plant contours.

### 3.2 Multi-scale adaptive feature pyramid network

The standard YOLOv11 neck adopts an FPN (Lin et al., 2017) structure in which multi-scale feature fusion is mainly implemented through upsampling and concatenation. Simple concatenation cannot adaptively regulate the contribution of features from different levels and does not explicitly account for their semantic differences. This limitation is particularly relevant for dense small-target weeds, where shallow spatial detail and deep semantics must interact effectively. MSAFPN addresses this issue by introducing the Attention-Guided Multi-Feature Aggregation Module (AMFAM) into a multi-level bidirectional fusion pathway. Adaptive weighted fusion is thereby used to improve representation of weed targets with different sizes and visual contexts. The structure of AMFAM is illustrated in **Figure 6**.

**Figure 6:**
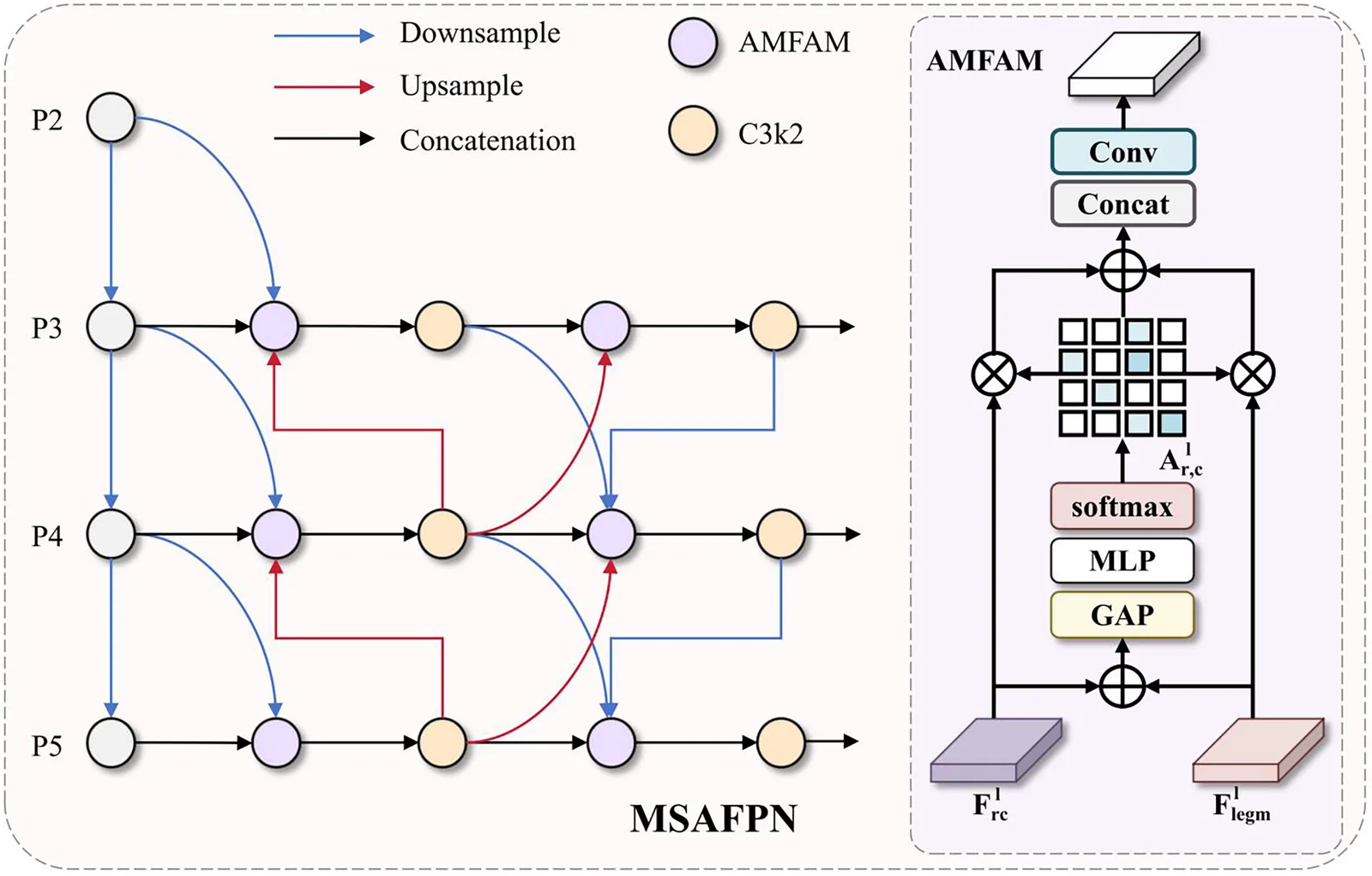
Architecture of the attention-guided multi-feature aggregation module (AMFAM).

The MSAFPN neck network adopts a multi-level bidirectional feature fusion architecture, with its core innovation being the replacement of simple concatenation operations with AMFAM and the construction of more complex cross-scale feature interaction pathways. The network first extracts multi-scale features from different backbone stages (P3, P4, P5) and then performs progressive feature fusion through a series of AMFAM modules. Unlike conventional YOLOv11, MSAFPN introduces additional cross-layer connections to enable shallow features to interact directly with deep features via intermediate layers. Specifically, in the top-down pathway, the network first fuses the deep semantic features from the P5 level, then propagates them upward to the P4 and P3 levels; in the bottom-up pathway, the refined features are propagated downward again and undergo secondary fusion with previous fusion results. This bidirectional multi-pathway feature flow mechanism ensures sufficient information exchange between features at different scales. The entire feature fusion process can be formulated as shown in Equation 4:

(4) $${ℱ}_{\text{out}}^{l}=\text{AMFAM}\left({ℱ}^{l},{ℱ}_{lsl}^{l},{ℱ}_{su}^{l}\right)⊙W_{t}^{l}+{ℱ}_{se}^{l}$$

where ${ℱ}_{out}^{l}$ denotes the fused feature at the *l*-th level; ${ℱ}^{l}$, ${ℱ}_{lsl}^{l}$, and ${ℱ}_{su}^{l}$ represent feature maps from the upper, lower, and cross-layer connections, respectively; $W_{t}^{l}$ is the adaptive weight matrix; ${ℱ}_{se}^{l}$ is the residual connection feature; and ⊙ denotes element-wise multiplication.

AMFAM realizes adaptive weighted fusion of multiple input features through a channel attention mechanism. The workflow of AMFAM comprises three key steps: feature alignment, attention generation, and weighted fusion. For multiple input feature maps with different channel counts, AMFAM first maps them to a unified channel dimension *C* via 1×1 convolutions to ensure that features reside in the same representation space. All aligned features are then concatenated along the channel dimension and reshaped into a tensor of shape (*B*, *N*, *C*, *H*, *W*), where *B* is the batch size, *N* is the number of input features, and *H* and *W* are the spatial dimensions. AMFAM then computes a preliminary fusion result ${ℱ}_{0}=\sum_{k=1}^{N}\phi_{k}\left({ℱ}_{k}\right)$ and compresses it into a global descriptor $\text{g}\in {\mathbb{R}}^{B\times C\times 1\times 1}$ via adaptive average pooling. This global descriptor is subsequently fed into a lightweight multilayer perceptron (MLP) to generate attention weights. The MLP consists of two convolutional layers that learn nonlinear relationships among features through dimensionality reduction and expansion; after Softmax normalization, the importance weight $\alpha_{k}\in {\mathbb{R}}^{B\times N\times C\times 1\times 1}$ of each feature map is obtained. The attention weight computation process can be formalized as shown in Equation 5:

(5) $$\alpha =\text{Softmax}\left(\text{MLP}\left(\text{GAP}\left(\sum_{k=1}^{N}\phi_{k}\left(F_{k}\right)\right)\right)\right)\;$$

where $\phi_{k}\left(\cdot \right)$ denotes the 1×1 convolution alignment operation, GAP(·) denotes global average pooling, and MLP(·) denotes the multilayer perceptron transformation. Finally, AMFAM generates the output feature by weighted summation as shown in Equation 6:

(6) $${ℱ}_{o}=\sum_{k=1}^{N}\alpha_{k}⊙\phi_{k}\left({ℱ}_{k}\right)$$

where *α_k_* is the normalized attention weight for the *k*-th feature and ⊙ denotes element-wise multiplication. This mechanism enables the network to autonomously learn the relative importance of features at different scales—assigning higher weights to deep features rich in semantic information while appropriately retaining shallow features rich in detailed information—thereby maintaining spatial localization precision while ensuring semantic understanding capability.

MSAFPN addresses some of YOLOv11’s weaknesses in weed detection problems with its adaptive keep-or-abandon feature fusion strategy and multi-pathway feature interaction architecture. In the particular fusion of adjacent layers, MSAFPN features degradation adaptively adjusted according to the semantic differences of features of different scales, which can solve the fixed-weight limitation of conventional FPN in handling multi-scale targets. This bidirectional multi-level feature flow mechanism encourages the effective fusion of the shallow detail features and deep semantic features. The more the network attends to the various shapes and their associated backgrounds of weeds, the more discriminative accuracy it has. The channel attention mechanism used in AMFAM allows great feature representation while keeping computation requirements low enough for edge deployment.

### 3.3 Enhanced channel shuffle upsampling module

The standard YOLOv11 uses fixed nearest-neighbor interpolation to increase feature-map resolution. Although this operation is computationally inexpensive, interpolation itself cannot recover high-frequency spatial information that has already been attenuated during previous down-sampling stages. Nearest-neighbour upsampling may also produce block-like local transitions, which can weaken the representation of fine target edges and textures during subsequent cross-scale fusion. ECSM is therefore designed to enhance local spatial representation after upsampling rather than to eliminate an artifact that is inherently caused by nearest-neighbour interpolation. The module combines depthwise convolution, channel shuffle, and spatial shift while maintaining a compact computational structure. Its architecture is illustrated in **Figure 7**.

**Figure 7:**
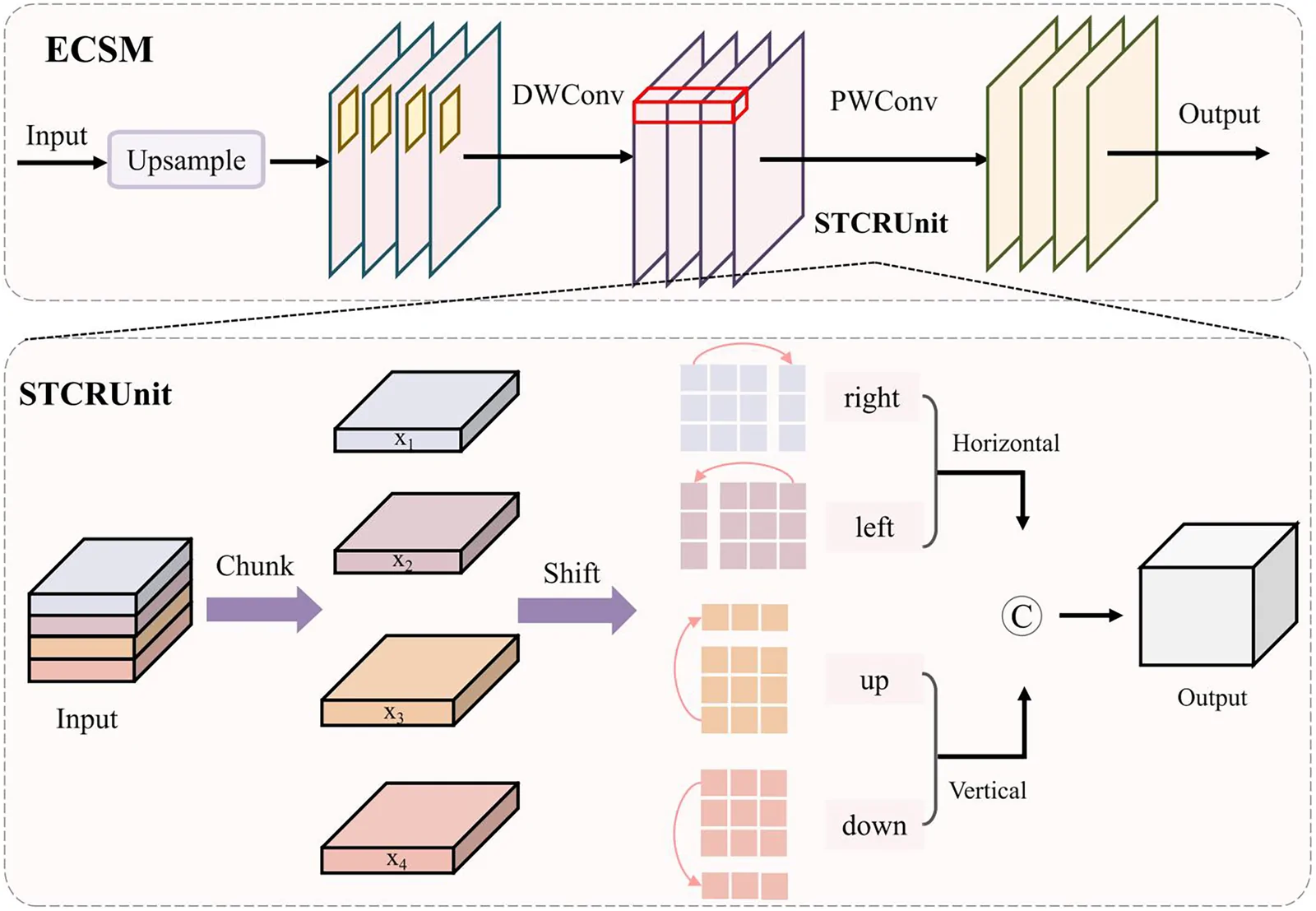
Architecture of the enhanced channel shuffle upsampling module (ECSM).

ECSM proposes a novel feature enhancement architecture that increases the feature map size via upsampling and incorporates depthwise separable convolution to balance complexity and feature extraction capability. The essence of the module is to build on the channel shuffle mechanism and then add lightweight spatial shift to enhance discriminability by reordering the patterns through which the inter-channel connection flows. The entire ECSM module can be mathematically expressed as shown in Equation 7:

(7) $$Y={ℱ}_{\text{pw}}\left(\text{STCRU}\left(ch\_sh\left({ℱ}_{\text{dw}}\left({↑}_{2}X\right)\right)\right)\right)$$

where ${↑}_{2}$ denotes 2× upsampling, ${ℱ}_{dw}$ represents depthwise separable convolution, *ch_sh* denotes the channel shuffle operation, STCRU is the Spatial-Temporal Channel Redistribution Unit, and ${ℱ}_{pw}$ represents pointwise convolution.

STCRU implements a parameter-free lightweight feature mixing strategy. The module first uniformly splits the input feature tensor into four sub-blocks along the channel dimension, and then applies cyclic shift operations in different spatial directions to each sub-block. Specifically, given input feature map $X\in {\mathbb{R}}^{C\times H\times W}$, the channel split operation is performed as shown in Equation 8:

(8) $$\left\{c_{1},c_{2},c_{3},c_{4}\right\}=\text{split}\left(X,\text{dim}=1\right)$$

where each sub-block $c_{i}\in {\mathbb{R}}^{C/4\times \text{H}\times \text{W}}$. Cyclic shift operations in different directions are then applied to each sub-block, as shown in Equation 9:

(9) $$\begin{array}{c} \begin{array}{c} c_{1}^{\prime }=roll\left(c_{1},s,dim=2\right)\\ c_{2}^{\prime }=roll\left(c_{2},-s,dim=2\right) \end{array}\\ \begin{array}{c} c_{3}^{\prime }=roll\left(c_{3},s,dim=3\right)\\ c_{4}^{\prime }=roll\left(c_{4},-s,dim=3\right) \end{array} \end{array}$$

where *s* is the shift step and roll(·) denotes the cyclic shift operation. By guiding different channel features to perceive different spatial positional information, this design achieves cross-channel local feature enhancement and spatial information redistribution, thereby improving the network’s feature representation capability. The features are finally recombined via channel concatenation, achieving efficient feature mixing.

The combined use of depthwise convolution, channel shuffle, and spatial shift enables ECSM to enhance spatial detail after fixed interpolation without attributing the improvement to removal of checkerboard artifacts. The module is intended to reduce information loss and block-like local transitions in the upsampled features, thereby improving subsequent multi-scale fusion and the representation of small weed targets in complex backgrounds.

## 4 Experiments

### 4.1 Public datasets

1. CottonWeedDet12 (Dang et al., 2023) is a multi-class weed detection dataset for use in cotton production systems in the southern United States, introduced by Dang et al. in 2023. The dataset consists of 5,648 images containing 12 distinct weed species and 9370 annotated bounding boxes. The images were collected using handheld smartphones or digital cameras at several research farms at Mississippi State University between June and September of 2021. Images were collected under a variety of weather conditions and stages of weed growth to ensure robustness of the models. All images were obtained in natural lighting. The dataset is publicly accessible on Zenodo and provides a standard dataset benchmark evaluation performed using 25 YOLO object detectors.
2. ImageWeeds (Rai et al., 2023) is an open-source weed image dataset by Rai et al. (2023) for real-time weed recognition and precision spraying applications. It consists of 3,975 images of five common weed species found in North Dakota: *Bassia scoparia, Ambrosia artemisiifolia, Conyza canadensis, Amaranthus retroflexus, Chenopodium album.* Dataset is innovatively split into 2 categories, “aerial weeds” and “individual weeds.” The aerial category contains 551 high-resolution drone images with 2,390 annotated weeds; the individual contains 3,424 single-plant weed images cropped from aerial and greenhouse images with 7,700 annotated instances. Annotations are available in TXT, XML, and JSON formats, ready for YOLO, Faster R-CNN, and other deep learning models.

### 4.2 Experimental setup

All experiments were run in similar software/hardware environments. Hardware consisted of an Intel Core i9-14900K, 64 GB DDR5 RAM, and NVIDIA GeForce RTX 4090 GPU. Software consisted of Windows 11, PyTorch 2.0.1 deep learning framework, CUDA 11.8 and cuDNN 8.6.0 for GPU accelerated computing, Python 3.10, YOLOv11 implementation based on the official Ultralytics open-source codebase, and OpenCV 4.8.0 for image processing.

Key training hyperparameters were as follows: input images were uniformly resized to 640×640 pixels; the maximum number of training epochs was 350 with a batch size of 32. Stochastic gradient descent (SGD) was used with an initial learning rate of 0.01, weight decay of 0.0005, and momentum of 0.937. Other training settings followed the default YOLOv11 configuration to maintain consistency with the baseline. Training used a single NVIDIA RTX 4090 GPU with Automatic Mixed Precision (AMP), and early stopping was triggered when validation mAP@0.5:0.95 failed to improve for 50 consecutive epochs. The group-wise training, validation, and test split described in Section 2.1 was fixed for all controlled comparisons. To assess training stability, the principal configurations were independently trained three times with different random seeds while keeping the data split, optimizer, schedule, and remaining hyperparameters unchanged.

### 4.3 Evaluation metrics

A multi-dimensional evaluation framework was used to assess Alfalfa-Yolo in terms of detection accuracy, model complexity, and inference efficiency.

Detection accuracy is evaluated using Precision (P), Recall (R), mAP@0.5, and mAP@0.5:0.95. Precision measures the proportion of predicted positive detections that are correct, whereas Recall measures the proportion of ground-truth targets that are successfully detected. mAP@0.5 evaluates average precision at an IoU threshold of 0.50, while mAP@0.5:0.95 averages AP over IoU thresholds from 0.50 to 0.95 in increments of 0.05. The latter therefore imposes progressively stricter bounding-box overlap requirements and provides a more demanding measure of localization quality within the bounding-box detection setting.

For model complexity, the following three metrics represent the scale of such model and computational burden. Parameters (Params, M): total number of trainable parameters in the model (million) so giving indication of storage resources needed and memory footprint of model. Model Size (MB): actual storage size of model weight file (megabytes). FLOPs (G): number of floating point operations needed during forward inference (billion), thereby giving indication of theoretical complexity of algorithm, so that smaller or fewer are preferred. Inference speed—Frames Per Second (FPS) directly measuring of number of image frames processed in a second in actually imaged on hardware.

### 4.4 Ablation study

#### 4.4.1 Ablation analysis of EPGTNet

To evaluate the contribution of the structures in EPGTNet, three groups of controlled ablations were conducted for the AMSEFG edge-feature generator, the PESFM multi-scale fusion configuration, and the placement of EPGTNet. AMSEFG ablation compares single-direction and bidirectional Sobel responses; PESFM ablation evaluates different feature-level combinations; and positional ablation examines backbone-only, neck-only, and combined placements. The results are reported in **Table 3** and **Figure 8**.

**Table 3:** Ablation experiment results for different components of EPGTNet.

Method	GFLOPs(G)	Params (M)	P(%)	R(%)	mAP@0.5 (%)	mAP@0.5:0.95 (%)	Size (MB)	FPS
AMSEFG Structural Ablation								
Sobel-*x* only	7.5	2.95	76.4	67.4	74.3	54.4	5.95	164.2
Sobel-*y* only	7.6	2.95	77.7	67.3	75.6	54.9	5.95	165.9
Sobel-*x*+*y*(Ours)	7.6	2.94	80.6	71.5	76.9	55.3	5.95	167.7
PESFM Structural Ablation								
PESFM-P3	7.2	2.65	78.8	66.0	74.1	54.4	5.39	188.5
PESFM-P4	6.7	2.65	79.9	66.2	73.2	53.2	5.39	191.2
PESFM-P5	6.7	2.82	77.0	69.9	75.2	54.5	5.72	171.7
PESFM-P3+P4	7.4	2.71	77.7	68.7	75.3	55.7	5.51	184.8
PESFM-P3+P5	7.4	2.88	74.9	71.1	75.7	55.2	5.80	183.3
PESFM-P4+P5	6.9	2.88	72.7	68.9	74.3	54.3	5.84	185.3
PESFM-P3+P4+P5 (Ours)	7.6	2.94	80.6	71.5	76.9	55.3	5.95	179.3
EPGTNet Positional Ablation								
EPGTNet-backbone only (Ours)	7.6	2.94	80.6	71.5	76.9	55.3	5.95	176.3
EPGTNet-neck only	8.3	3.25	78.8	68.8	76.0	54.2	6.55	153.0
EPGTNet-backbone+neck	9.6	3.43	78.5	67.9	75.7	56.2	6.92	162.7

**Figure 8:**
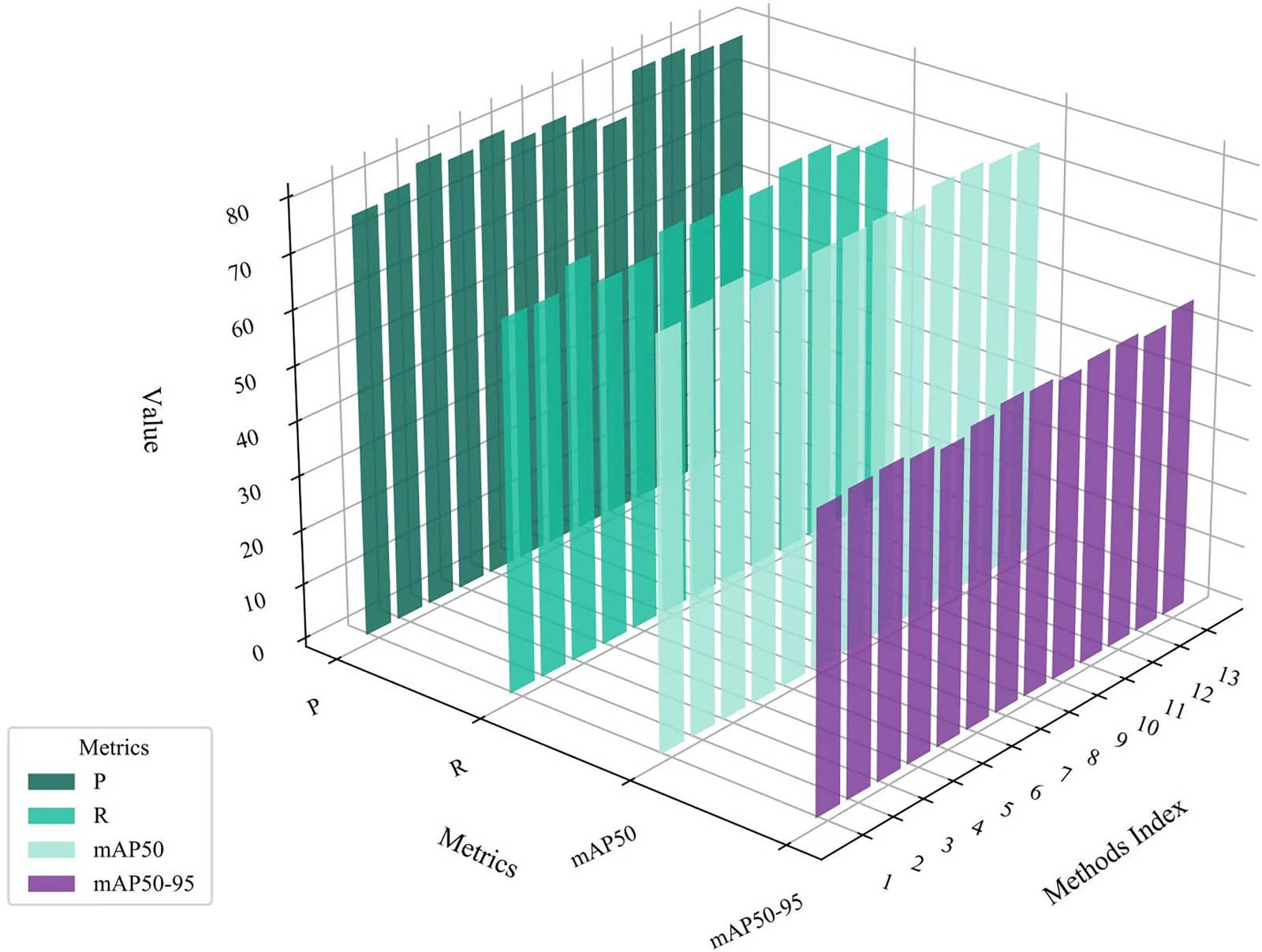
Comparative results of ablation experiments on different EPGTNet components. Labels on the *x*-axis: 1: Sobel-*x* only; 2: Sobel-*y* only; 3: Sobel-*x*+*y* (Ours); 4: PESFM-P3; 5: PESFM-P4; 6: PESFM-P5; 7: PESFM-P3+P4; 8: PESFMP3+P5; 9: PESFM-P4+P5; 10: PESFM-P3+P4+P5 (Ours); 11: EPGTNet-backbone only (Ours); 12: EPGTNet-neck only; 13: EPGTNet-backbone+neck.

The bidirectional Sobel configuration in AMSEFG provides the strongest overall result among the directional variants. Sobel-x+y raises Recall to 71.5%, with mAP@0.5 and mAP@0.5:0.95 reaching 76.9% and 55.3%, respectively. At the bounding-box level, the increase in mAP@0.5:0.95 is particularly relevant because this metric averages performance over IoU thresholds from 0.50 to 0.95. Relative to the YOLOv11n baseline values of 74.4% mAP@0.5 and 53.9% mAP@0.5:0.95, introducing EPGTNet improves the two metrics by 2.5 and 1.4 percentage points, indicating that the gain is retained under stricter overlap requirements rather than being limited to IoU = 0.50. This evidence supports the use of boundary-related gradient cues for box-level localization, but it is not interpreted as pixel-level contour accuracy. For PESFM, the P3+P4+P5 configuration achieves the most balanced multi-level result, while the backbone-only EPGTNet placement retains competitive detection accuracy with 2.94 M parameters and 176.3 FPS.

#### 4.4.2 Ablation analysis of MSAFPN

To verify the effectiveness of the AMFAM module within MSAFPN, systematic ablation experiments were conducted covering the individual use of P3, P4, and P5 feature levels and three two-level combination schemes (P3+P4, P3+P5, P4+P5), with results compared against the proposed three-level complete fusion scheme AMFAM-P3+P4+P5. Results are presented in **Table 4**.

**Table 4:** Ablation experiment results for the AMFAM multi-scale adaptive feature fusion module within MSAFPN.

Model	GFLOPs(G)	Params(M)	P(%)	R(%)	mAP@0.5(%)	mAP@0.5:0.95(%)	Size(MB)
AMFAM Structural Ablation							
AMFAM-P3	6.9	2.68	79.3	66.6	74.2	55.6	5.49
AMFAM-P4	6.9	2.65	76.8	66.2	73.6	54.9	5.42
AMFAM-P5	7.0	2.65	72.2	70.5	75.5	55.9	5.43
AMFAM-P3+P4	6.8	2.64	80.7	66.8	74.4	54.1	5.41
AMFAM-P3+P5	6.9	2.64	74.8	65.8	72.4	52.7	5.42
AMFAM-P4+P5	6.9	2.61	82.3	63.9	74.9	55.6	5.35
AMFAM-P3+P4+P5 (Ours)	6.7	2.60	79.9	66.6	75.6	54.8	4.56

The experimental results reveal that different feature levels exhibit complementary detection characteristics: the P3 level focuses on capturing shallow detail features, achieving a precision of 79.3% but recall of only 66.6%; the P5 level concentrates on deep semantic information and improves recall by 3.9% over P3, indicating that deep features help reduce missed detections; and the P4 level achieves a good balance between precision and recall as an intermediate level. Among two-level fusion schemes, the P4+P5 combination achieves the best performance, with precision improving by 3.0% over the best single-level scheme, benefiting from the synergistic effect of mid-to-high-level features on both target localization precision and semantic discriminability. Specifically, the proposed three-level thorough fusion scheme AMFAM-P3+P4+P5 achieves the best global performance while maintaining the competitive merit of being lightweight, with mAP@0.5 and mAP@0.5:0.95 increasing by 0.7% compared to the best two-level scheme. By adaptively fusing the shallow fine-grained texture features with other mid-level position-sensitive features from the middle and deep high-level semantic features, the three-level thorough scheme provides a cooperative multi-scale feature representation mechanism to improve the detection generalization capability on weed targets of diverse growing cycles and scales.

#### 4.4.3 Overall ablation study

To evaluate the independent and combined effects of the three core components, an overall ablation study was conducted using YOLOv11n as the baseline. EPGTNet, MSAFPN, and ECSM were tested individually, in pairwise combinations, and in the complete Alfalfa-Yolo configuration. The results are summarized in **Table 5** and **Figure 9**.

**Table 5:** Overall ablation experiment results for Alfalfa-Yolo.

Config	EPGTNet	MSAFPN	ECSM	GFLOPs (G)	Params (M)	P (%)	R (%)	mAP@0.5 (%)	mAP@0.5:0.95 (%)
1. Baseline				6.4	2.59	79.2	68.8	74.4	53.9
2	✓			7.6	2.94	80.6	71.5	76.9	55.3
3		✓		6.7	2.61	79.9	66.6	75.6	54.8
4			✓	6.4	2.24	78.3	68.6	76.5	55.3
5	✓	✓		8.0	2.96	77.4	71.0	77.2	55.8
6	✓		✓	7.7	2.88	83.3	65.9	75.7	55.4
7		✓	✓	6.8	2.31	82.4	66.8	76.2	55.1
8. Ours	✓	✓	✓	8.1	2.67	80.4	70.9	77.6	55.9

**Figure 9:**
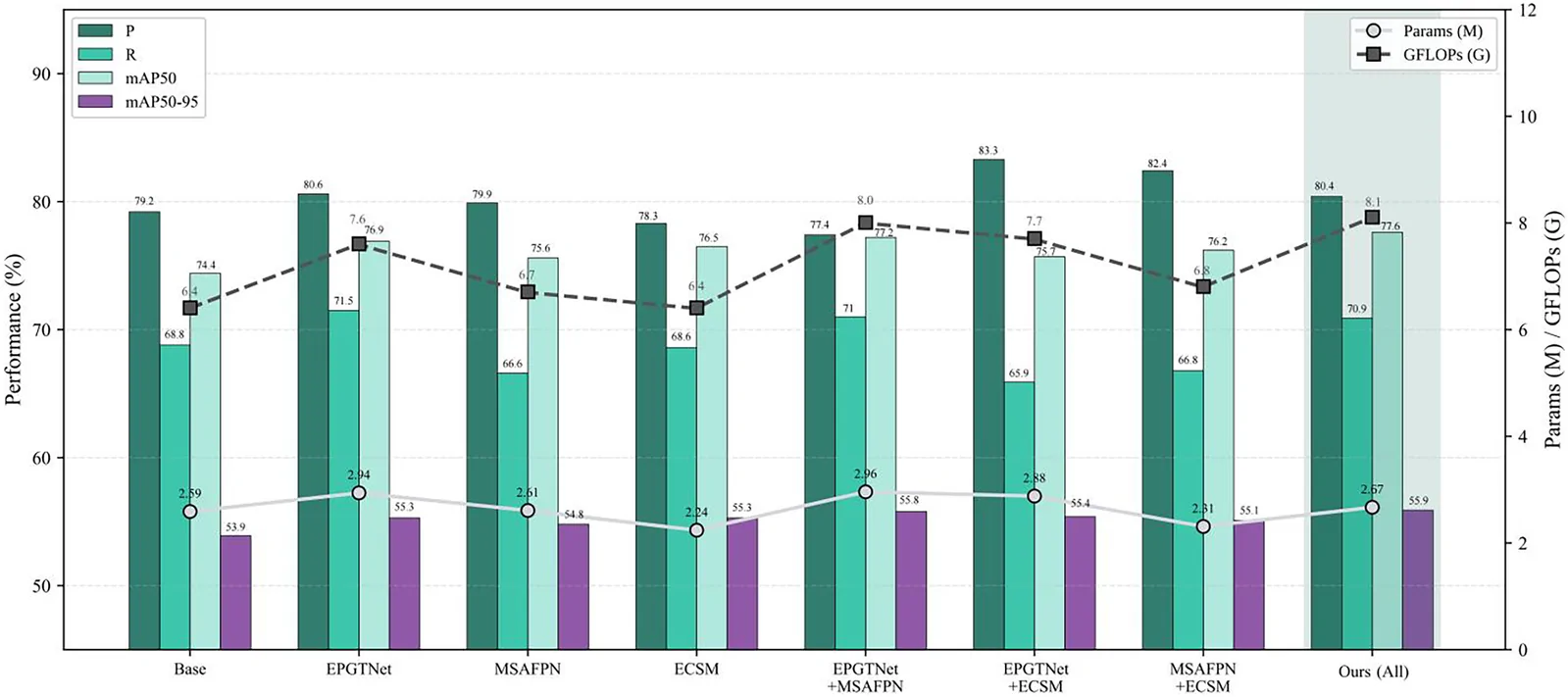
Comparative results of overall ablation experiments.

The experimental results verify the effectiveness of each of the three core modules independently. EPGTNet with mAP@0.5 increased by 2.5% and recall with a 2.7% gain over the baseline, as the improved convolutional structure captures more high-level multi-scale spatial features of weed targets, thereby achieving a much better perception of the “small weed” targets hidden among complicated backgrounds. The MSAFPN has a slightly lower recall but improves mAP@0.5:0.95 by 0.9% with a reduction of 12.9% in model size due to its adaptive feature fusion strategy, thereby highlighting its advantage of maintaining detection accuracy while being lightweight. ECSM attention module generates a 2.1% gain in mAP@0.5 with the least parameters through its dual-dimensional channel and spatial attention pump, adaptively enhancing areas of interest while suppressing non-informative content. In dual-module combinations, the unique synergistic effect of the EPGTNet + ECSM combination, with precision improving by 4.1% over the baseline, indicates that the rich features extracted by the improved backbone achieve great gains in detection confidence and localization precision after passing through the “funnel effect” of the attention mechanism that carefully “filters out” noise. The organic fusion of the three core modules in the complete Alfalfa-Yolo achieves the best balance of accuracy, recall, and model complexity—the mAP@0.5 is improved by 3.2% and mAP@0.5:0.95 improves by 2.0% over the baseline, with 9.8% fewer parameters than the best dual-module scheme. The deep synergy of EPGTNet’s multi-scale feature extraction, MSAFPN’s richer representation of features from context, and ECSM’s “pumping” of features can be said to construct a complete detection pipeline from low-level feature extraction to high-level semantic understanding, fully validating the complementarity of the modules and the effectiveness of the architecture itself.

Grad-CAM is used only to visualize relative spatial response patterns and is not treated as causal evidence of module effectiveness, boundary accuracy, or cross-domain generalization. The four displayed examples were selected from the predefined test set to cover different target scales, weed densities, and background complexities, and identical input images were used for all compared configurations. **Figure 10** therefore provides a qualitative complement to the quantitative ablation results.

**Figure 10:**
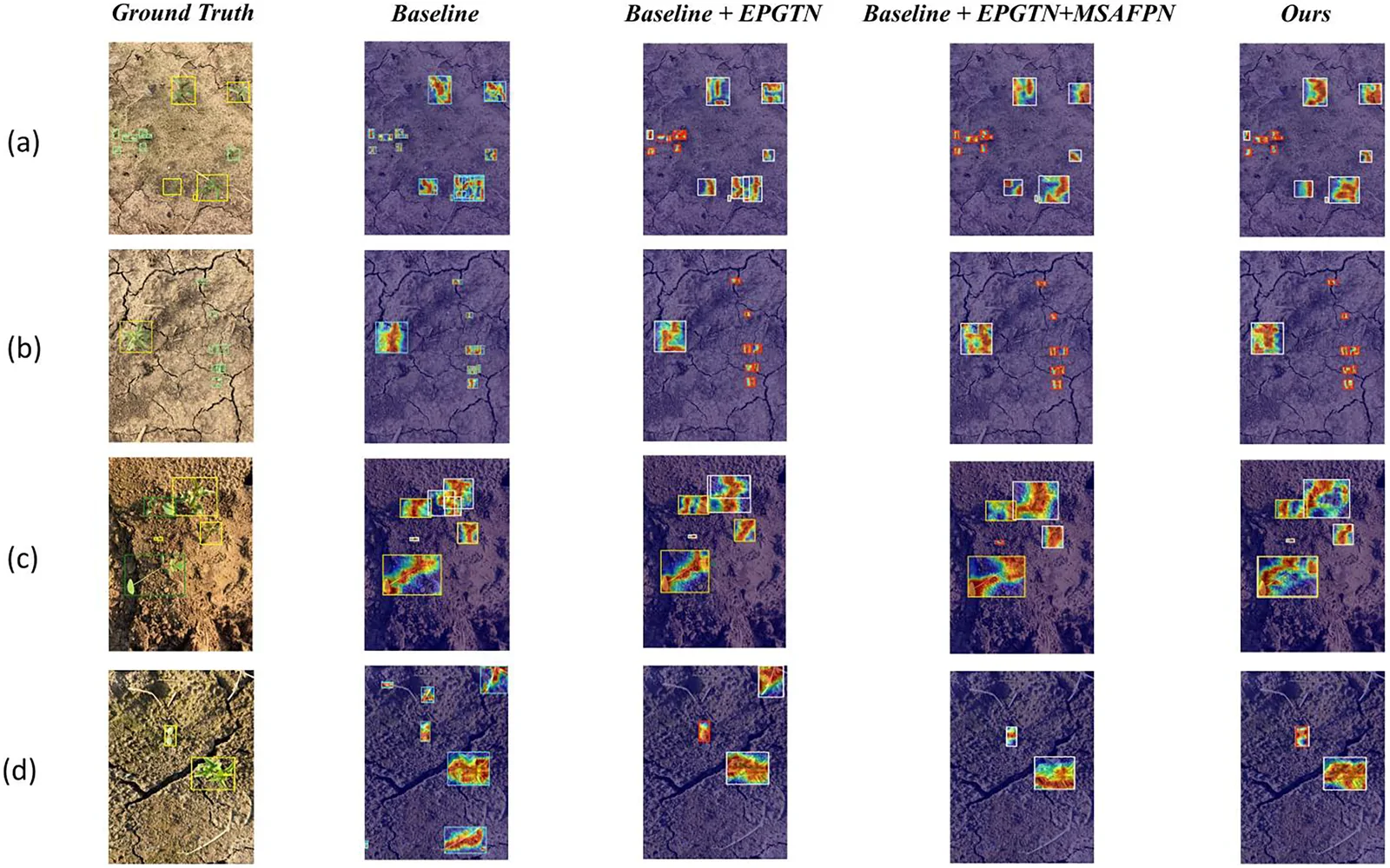
Grad-CAM visualization for the ablation study. Columns from left to right show Ground Truth, Baseline, Baseline + EPGTNet, Baseline + EPGTNet + MSAFPN, and Alfalfa-Yolo (Ours). Rows **(a–d)** correspond to four representative field samples.

The heatmaps show that the spatial response pattern changes as the modules are introduced. The baseline exhibits relatively dispersed activation in several cluttered samples, whereas EPGTNet and the subsequent modules concentrate a larger portion of the response around weed regions in the displayed cases. These visual differences are interpreted only as qualitative feature-response behavior. They are not used as quantitative proof of boundary fidelity, localization accuracy, or a causal interaction among modules; the corresponding performance conclusions are based on the detection metrics reported in **Table 5** and the repeated-run analysis below.

#### 4.4.4 Stability analysis

To evaluate whether the reported gains are robust to training randomness, the principal configurations were independently trained three times using different random seeds while the group-wise data split, training schedule, optimizer, and all other hyperparameters were held constant. The repeated experiments cover the YOLOv11n baseline, the three principal single-module configurations, the complete Alfalfa-Yolo, and the main independent-dataset benchmarks. Mean values, standard deviations, and 95% confidence intervals are reported in **Table 6**.

**Table 6:** Stability of the principal configurations across three independent runs.

Dataset/configuration	mAP@0.5 mean ± std	95% CI	mAP@0.5:0.95 mean ± std	95% CI
Self dataset, YOLOv11n	74.38 ± 0.17	73.96 to 74.80	53.88 ± 0.12	53.58 to 54.18
Self dataset, EPGTNet	76.87 ± 0.19	76.40 to 77.34	55.28 ± 0.14	54.93 to 55.63
Self dataset, MSAFPN	75.58 ± 0.16	75.18 to 75.98	54.80 ± 0.11	54.53 to 55.07
Self dataset, ECSM	76.47 ± 0.20	75.97 to 76.97	55.27 ± 0.13	54.95 to 55.59
Self dataset, Alfalfa-Yolo	77.55 ± 0.18	77.10 to 78.00	55.87 ± 0.12	55.57 to 56.17
CottonWeedDet12, YOLOv11n	93.38 ± 0.14	93.03 to 93.73	88.02 ± 0.10	87.77 to 88.27
CottonWeedDet12, Alfalfa-Yolo	94.85 ± 0.11	94.58 to 95.12	88.87 ± 0.09	88.65 to 89.09
ImageWeeds, YOLOv11n	74.20 ± 0.22	73.65 to 74.75	45.83 ± 0.17	45.41 to 46.25
ImageWeeds, Alfalfa-Yolo	78.47 ± 0.19	78.00 to 78.94	49.30 ± 0.15	48.93 to 49.67

The repeated-run results show limited dispersion across the principal configurations. On the self-constructed dataset, mAP@0.5 standard deviations range from 0.16 to 0.20 percentage points, while the complete model reaches 77.55 ± 0.18% compared with 74.38 ± 0.17% for YOLOv11n. The corresponding mAP@0.5:0.95 values are 55.87 ± 0.12% and 53.88 ± 0.12%, respectively. The same pattern is retained on CottonWeedDet12 and ImageWeeds after target-dataset retraining. These results indicate that the main accuracy differences are not attributable to a single favorable random initialization and provide an explicit uncertainty range for the reported improvements.

### 4.5 Comparative experiments

#### 4.5.1 Backbone comparison

To compare EPGTNet with representative alternatives, four C3k2-related schemes were evaluated under the same training and evaluation settings: FasterSFSC, EfficientVIM, SWC, and LFEM. The controlled comparison is reported in **Table 7**.

**Table 7:** Comparison results for different C3k2 variants.

Model	GFLOPs (G)	Params (M)	P (%)	R (%)	mAP@0.5 (%)	mAP@0.5:0.95 (%)	Size (MB)
C3k2 (base)	6.4	2.59	79.2	68.8	74.4	53.9	5.24
C3k2-FasterSFSC (Chen et al., 2023)	5.8	4.91	76.8	66.8	72.4	53.2	2.32
C3k2-EfficientVIM (Lee et al., 2025)	6.7	5.09	75.9	67.4	73.3	53.1	2.47
C3k2-SWC (Li et al., 2024)	8.1	6.55	78.2	64.7	72.9	52.9	3.28
C3k2-LFEM (Lu et al., 2025)	9.2	5.16	85.3	63.4	74.3	54.3	2.50
EPGTNet (Ours)	7.6	2.94	80.6	71.5	76.9	55.3	5.95

The results in **Table 7** show clear accuracy-efficiency trade-offs among the alternative backbone designs. FasterSFSC reduces computation but loses mAP@0.5, whereas EfficientVIM and SWC introduce additional complexity without producing a consistent gain across detection metrics. LFEM achieves high precision but lower recall. EPGTNet reaches 76.9% mAP@0.5 and 55.3% mAP@0.5:0.95 with 2.94 M parameters, providing the strongest aggregate detection result among the compared C3k2 variants while retaining moderate model complexity.

#### 4.5.2 Feature pyramid comparison

To compare MSAFPN with representative feature-pyramid alternatives, RFPN, WFU, DPCF, and HS-FPN were evaluated using identical backbone, detection-head, training, and evaluation settings. Results are presented in **Table 8**.

**Table 8:** Comparison results for different feature pyramid network architectures.

Model	GFLOPs (G)	Params (M)	P (%)	R (%)	mAP@0.5 (%)	mAP@0.5:0.95 (%)	Size (MB)
PAFPN (base)	6.4	2.59	79.2	68.8	74.4	53.9	5.24
RFPN (Li, 2024)	5.8	5.22	80.7	63.9	73.6	52.5	2.52
WFU (Li et al., 2024)	6.5	5.25	81.7	66.8	74.7	54.6	2.58
DPCF (Xu et al., 2025)	7.4	5.44	74.4	69.5	74.3	55.2	2.68
HS-FPN (Shi et al., 2025)	8.6	6.65	77.3	63.9	72.7	52.3	3.30
MSAFPN (Ours)	6.7	2.61	79.9	66.6	75.6	54.8	4.56

The experimental results show that there are significant differences in performance between the FPNs explored. RFPN improves feature propagation by a recursive mechanism, where mAP@0.5 improves by 1.5%, but recall drops by 4.9%, limiting detection ranges. WFU uses a weighted fusion strategy and improves mAP@0.5:0.95, but reaches 0.7%. Increasing parameters to 5.25M limits the efficiency of edge deployment. DPCF builds rich channels for feature interactions across dense connections. Thus, the increase in recall to 69.5% indicates that more complex patterns of connection lead to a situation where an abundance of information creates redundancy. HS-FPN’s hierarchical selective fusion mechanism is below the baseline on all metrics, with mAP@0.5 decreased by 1.7%, possibly because the selective strategy has difficulty providing the optimal learned weights for filtering features. MSAFPN achieves the best comprehensive performance with moderate computational complexity; mAP@0.5 improves by 1.2%, mAP@0.5:0.95 improves by 0.9%, and it achieves a good balance between precision and recall.

#### 4.5.3 Overall comparison

A broader comparison was conducted across two-stage detectors, Transformer-based end-to-end detectors, YOLO-series models, and other recent one-stage detectors using the same training and test sets, augmentation procedures, and evaluation metrics. Results are presented in **Table 9** and **Figure 11**.

**Table 9:** Overall performance comparison of Alfalfa-Yolo against mainstream object detection algorithms.

Model	GFLOPs (G)	Params (M)	P (%)	R (%)	mAP@0.5 (%)	mAP@0.5:0.95 (%)	Size (MB)	FPS
Two-Stage Object Detectors								
Faster R-CNN (Ren et al., 2015)	370	137	41.9	66.8	55.4	24.8	108	45.45
SSD-MobileNetV2 (Liu et al., 2016)	6.2	3.8	78.1	65.7	41.4	17.7	15.3	164.2
Transformer-Based Object Detectors								
DETR (Carion et al., 2020)	38.3	41.5	78.4	58.9	58.8	40.8	474	51.8
RT-DETR-R18 (Zhao et al., 2024)	56.9	19.8	82.1	70.2	74.3	53.5	76.9	76.3
One-Stage Object Detectors								
YOLOv5n (Jocher et al., 2020)	5.8	2.18	77.7	64.6	73.9	52.8	4.45	226.83
YOLOv6n (Li et al., 2022)	11.5	4.15	79.5	65.4	73.4	53.4	8.17	255.1
YOLOv8n (Jocher et al., 2023)	6.8	2.68	76.7	66.7	73.9	52.6	5.4	244.2
YOLOv10n (Wang et al., 2024)	6.5	2.26	79.5	64.7	72.0	51.4	5.51	209.91
YOLOv12n (Tian et al., 2025)	5.8	2.51	75.0	67.6	73.3	52.6	5.21	127.35
YOLOv13n (Lei et al., 2025)	6.1	2.44	77.7	65.8	73.8	53.6	5.17	90.49
Hyper-YOLO (Feng et al., 2024)	9.5	3.62	76.1	68.5	73.8	52.0	7.28	152.22
YOLOv11n (Baseline)	6.4	2.59	79.2	68.8	74.4	53.9	5.24	210.63
Alfalfa-Yolo (Ours)	8.1	2.67	80.4	70.9	77.6	55.9	5.5	178.68

**Figure 11:**
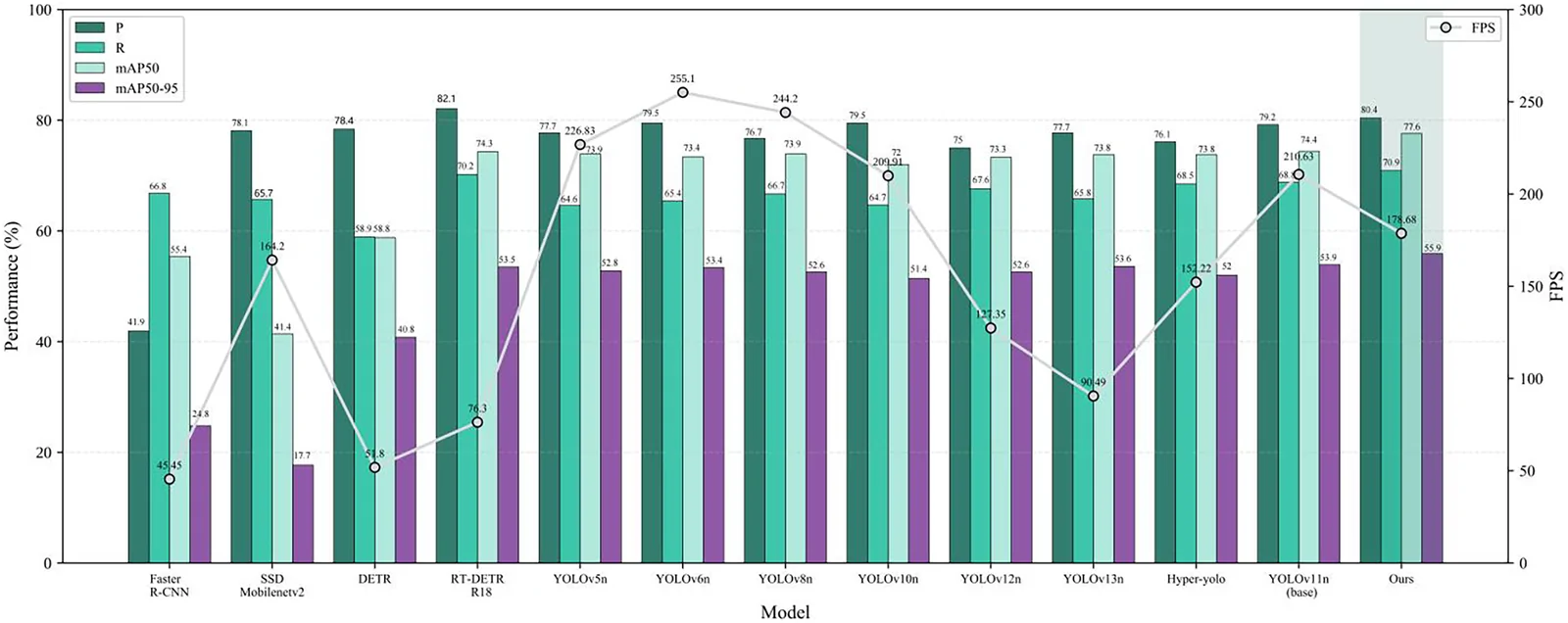
Overall comparison results between Alfalfa-Yolo and mainstream object detection algorithms.

**Table 9** reveals substantial trade-offs between detection accuracy and computational efficiency. Faster R-CNN requires 370 GFLOPs and 137 M parameters with 45.45 FPS, while RT-DETR-R18 reaches 53.5% mAP@0.5:0.95 at 56.9 GFLOPs and 76.3 FPS. YOLOv6n provides the highest throughput among the compared one-stage models but with lower recall and mAP@0.5. Alfalfa-Yolo achieves 80.4% precision, 70.9% recall, 77.6% mAP@0.5, and 55.9% mAP@0.5:0.95. Relative to YOLOv11n, however, the parameter count increases from 2.59 M to 2.67 M and computational cost increases from 6.4 to 8.1 GFLOPs, while RTX 4090 throughput decreases from 210.63 to 178.68 FPS. The proposed model is therefore not uniformly lighter than YOLOv11n across all efficiency metrics. Instead, it retains a compact parameter scale of approximately 2.7 M while introducing additional computation in exchange for higher detection accuracy. Compared with substantially larger detectors such as Faster R-CNN and RT-DETR-R18, it still maintains a relatively compact complexity profile.

#### 4.5.4 Visualization results

For qualitative comparison, four representative field samples containing alfalfa seedlings, alfalfa branches, and weeds were selected from the predefined test set, and identical images were used for YOLOv11n, YOLOv13, and Alfalfa-Yolo. The detection results are shown in **Figure 12**.

**Figure 12:**
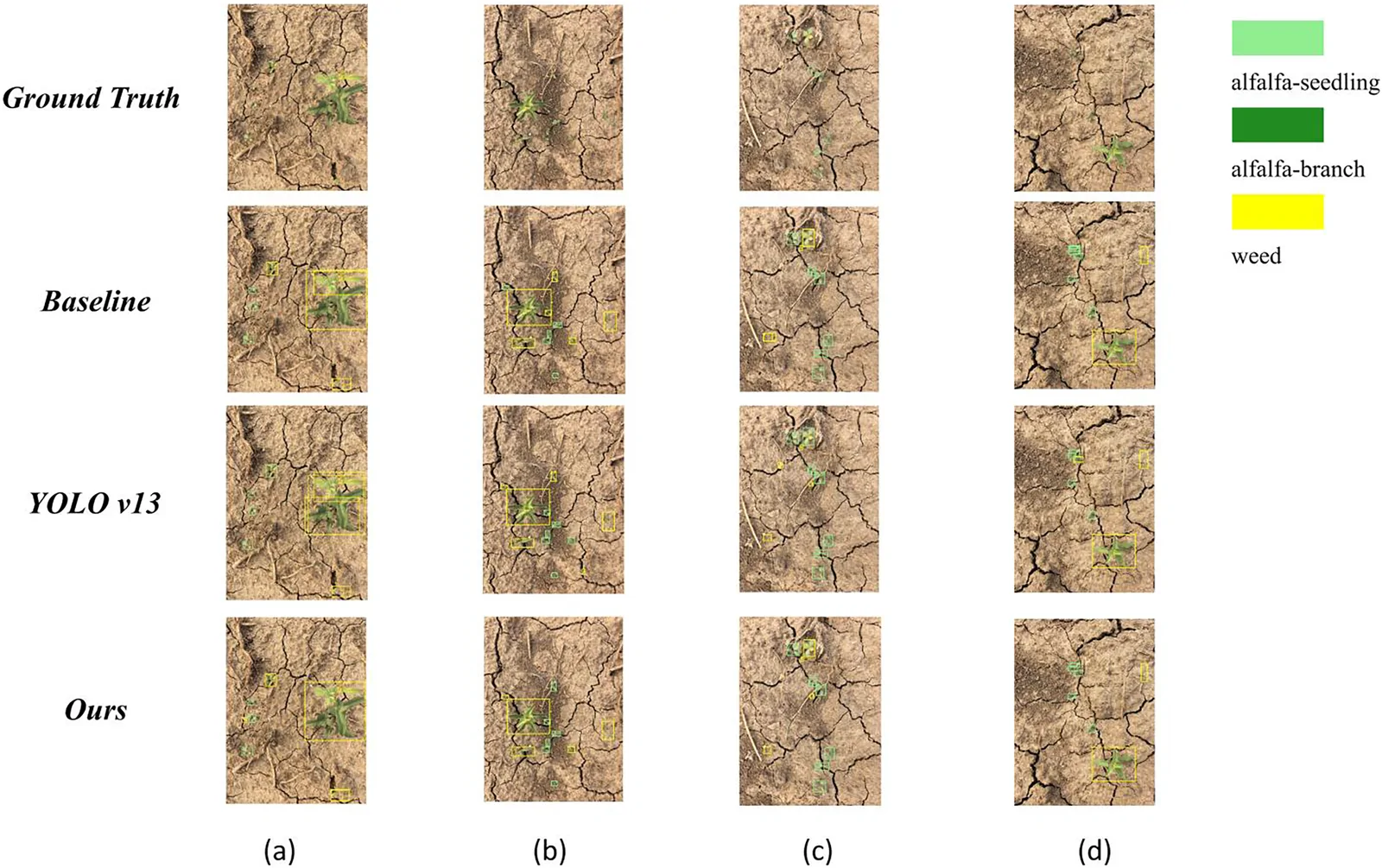
Visualization comparison of detection results. Rows from top to bottom show Ground Truth, Baseline, YOLOv13, and Alfalfa-Yolo (Ours). Columns **(a–d)** correspond to four representative field samples.

The visualization results demonstrate that Alfalfa-Yolo exhibits clear advantages over Baseline and YOLOv13 in target detection completeness, category discriminability, and small-target response capability. The features miss weed targets in the background in the Baseline samples (c) and (d), with localization offsets for the alfalfa seedlings and branches, indicating that the labeled features have insufficient discriminative capability in terms of multi-class coexistence and large target scale differences. YOLOv13 has better detection frame coverage of the alfalfa branches in sample (b) but has partially misdetected targets and oversized detection frames in samples (c) and (d). Alfalfa-Yolo, on the other hand, successfully detects all three types of targets in all 4 of the target samples, with its best improvement in locating densely distributed fine weed individuals in sample (d) and the most overlap between the detection frames and the targets.

### 4.6 Cross-dataset evaluation

#### 4.6.1 Independent-dataset benchmarking

To assess whether the proposed architecture remains effective under different public weed datasets, independent-dataset benchmarking was conducted on CottonWeedDet12 and ImageWeeds. Every compared detector was independently trained using the training split of the corresponding target dataset and then evaluated on its test split under the same training strategy and augmentation settings. These experiments therefore measure cross-dataset applicability after target-dataset training and are not interpreted as zero-shot cross-domain generalization. Results are presented in **Table 10** and **Figure 13**.

**Table 10:** Independent-dataset benchmarking results of Alfalfa-Yolo and comparison models.

Model	GFLOPs (G)	Params (M)	P (%)	R (%)	mAP@0.5 (%)	mAP@0.5:0.95 (%)	Size (MB)
Dataset 1: CottonWeedDet12							
YOLOv5n	5.8	2.18	91.0	88.0	93.2	88.0	4.45
YOLOv8n	6.8	2.68	91.6	86.0	93.6	88.2	5.38
YOLOv10n	6.5	2.26	91.8	83.6	92.0	86.5	5.50
YOLOv12n	5.8	2.51	93.3	88.0	94.7	89.4	5.21
YOLOv13n	6.1	2.45	94.8	85.2	94.4	89.4	5.17
YOLOv11n	6.3	2.58	94.5	84.3	93.4	88.0	5.23
Alfalfa-Yolo (Ours)	8.1	2.67	90.1	88.5	94.9	88.9	5.52
Dataset 2: ImageWeeds							
YOLOv5n	5.8	2.18	64.8	67.5	72.2	43.2	4.45
YOLOv8n	6.8	2.68	75.5	66.5	73.2	44.0	5.39
YOLOv10n	6.5	2.26	72.8	69.7	76.6	49.3	5.51
YOLOv12n	5.8	2.51	74.5	73.7	78.1	49.3	5.21
YOLOv13n	6.1	2.44	80.4	66.9	76.7	48.1	5.18
YOLOv11n	6.3	2.58	73.4	66.4	74.2	45.8	5.24
Alfalfa-Yolo (Ours)	8.1	2.67	69.6	74.3	78.5	49.3	5.53

**Figure 13:**
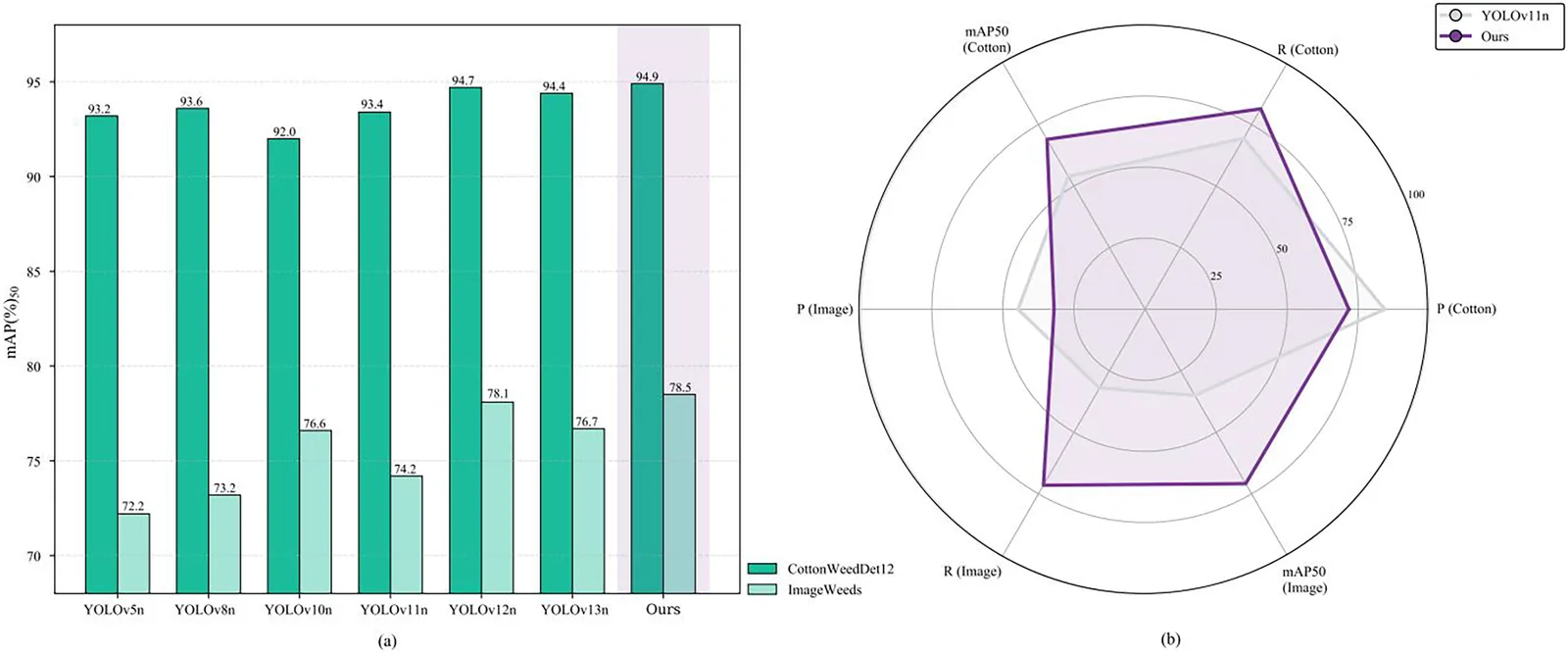
Independent-dataset benchmarking results on public weed datasets. Left: CottonWeedDet12; right: ImageWeeds.

The independently retrained results in **Table 10** show that Alfalfa-Yolo remains competitive under both public dataset distributions. On CottonWeedDet12, the model reaches 94.9% mAP@0.5 and 88.9% mAP@0.5:0.95, while Recall increases to 88.5%. On ImageWeeds, Alfalfa-Yolo reaches 78.5% mAP@0.5 and 49.3% mAP@0.5:0.95 with 74.3% Recall. Because all models are retrained on the corresponding target datasets, these gains indicate that the architecture can be effectively optimized under different crop backgrounds, imaging conditions, and weed compositions; they do not demonstrate source-only cross-domain transfer. The single generic weed label is retained as a localization task and these results are not interpreted as species-level recognition capability.

#### 4.6.2 Strict cross-dataset transfer

A strict cross-dataset transfer protocol was further used to evaluate performance without target-domain training. YOLOv11n and Alfalfa-Yolo were trained exclusively on the self-constructed alfalfa dataset, after which all model parameters were frozen and the models were directly evaluated on CottonWeedDet12 and ImageWeeds. No target-domain training, fine-tuning, model selection, or threshold optimization was performed. For label-space consistency, all weed categories in the target datasets were mapped to a generic weed category only during evaluation. **Table 11** reports the resulting source-only transfer performance.

**Table 11:** Strict zero-shot cross-dataset transfer from the self-constructed alfalfa dataset.

Source training set	Target test set	Model	mAP@0.5	mAP@0.5:0.95
Alfalfa	CottonWeedDet12	YOLOv11n	46.2 ± 0.14	26.0 ± 0.09
Alfalfa	CottonWeedDet12	Alfalfa-Yolo	50.1 ± 0.11	28.8 ± 0.04
Alfalfa	ImageWeeds	YOLOv11n	39.7 ± 0.16	21.9 ± 0.12
Alfalfa	ImageWeeds	Alfalfa-Yolo	43.8 ± 0.15	24.4 ± 0.08

The zero-shot results are substantially lower than the target-dataset retraining results in **Table 10**, directly reflecting the domain shift associated with different crop backgrounds, imaging devices, lighting conditions, and weed appearance distributions. Nevertheless, Alfalfa-Yolo retains a consistent advantage over YOLOv11n in both target domains. On CottonWeedDet12, mAP@0.5 increases from 46.2 ± 0.14% to 50.1 ± 0.11% and mAP@0.5:0.95 from 26.0 ± 0.09% to 28.8 ± 0.04%. On ImageWeeds, the corresponding values increase from 39.7 ± 0.16% to 43.8 ± 0.15% and from 21.9 ± 0.12% to 24.4 ± 0.08%. These source-only results are used to characterize cross-domain transfer behavior, whereas the independently retrained results are reported separately as evidence of cross-dataset applicability.

#### 4.6.3 Cross-dataset visualization

Qualitative visualization was conducted on the same predefined CottonWeedDet12 and ImageWeeds samples for the Baseline and Alfalfa-Yolo, as shown in **Figures 14** and **15**. The examples were selected to cover different target scales and background conditions, and the same underlying samples were retained for all compared models to avoid *post hoc* selection of visually favorable cases. Grad-CAM is used only to visualize relative spatial response patterns and is not treated as evidence of causal module effects, boundary fidelity, or cross-domain generalization.

**Figure 14:**
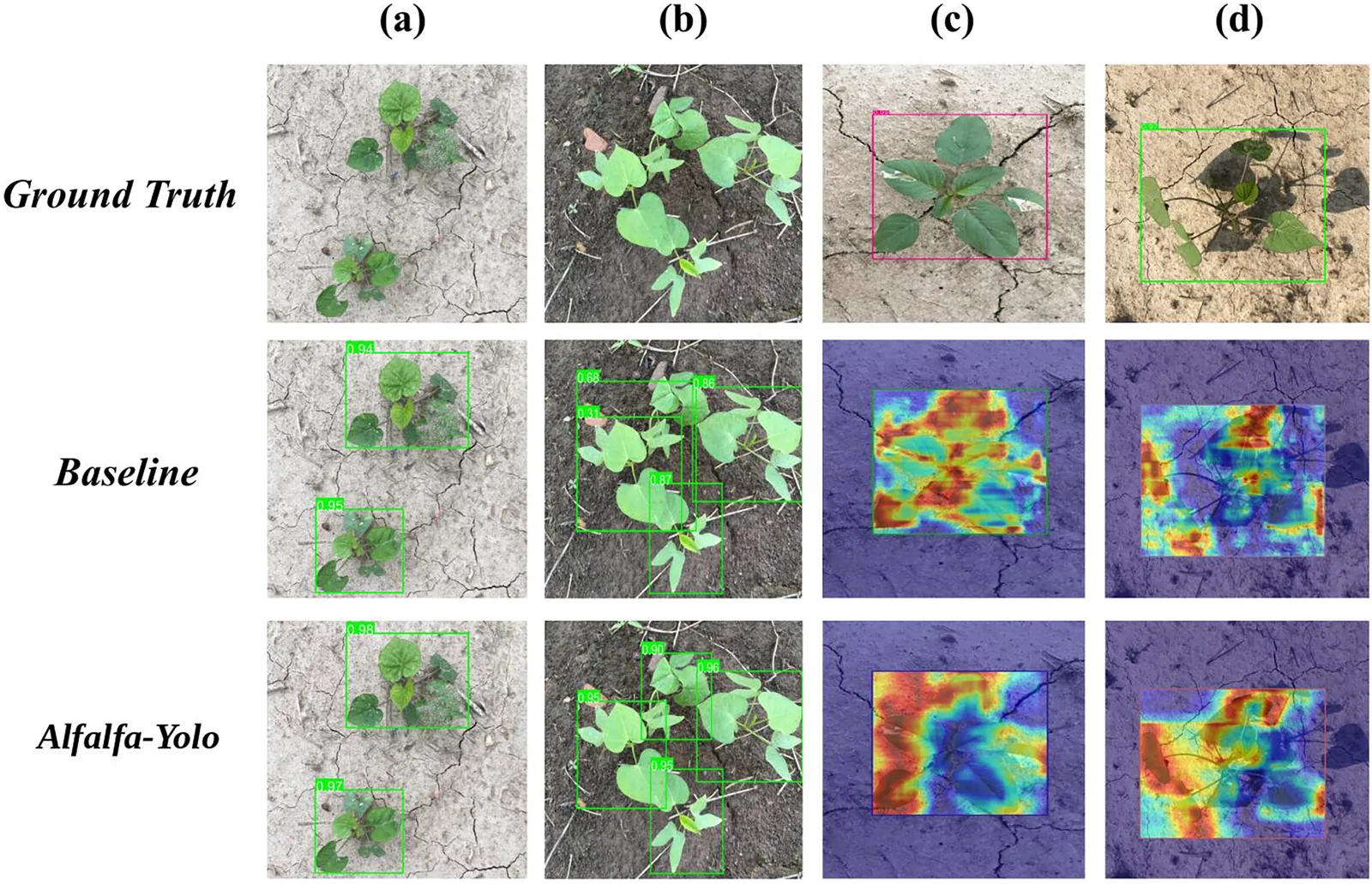
Comparison of the Baseline and Alfalfa-Yolo on representative CottonWeedDet12 samples. Columns **(a, b)** present bounding-box detection examples, whereas columns **(c, d)** present Grad-CAM response examples. Warmer colors indicate relatively stronger model responses and cooler colors indicate weaker responses within each image.

**Figure 15:**
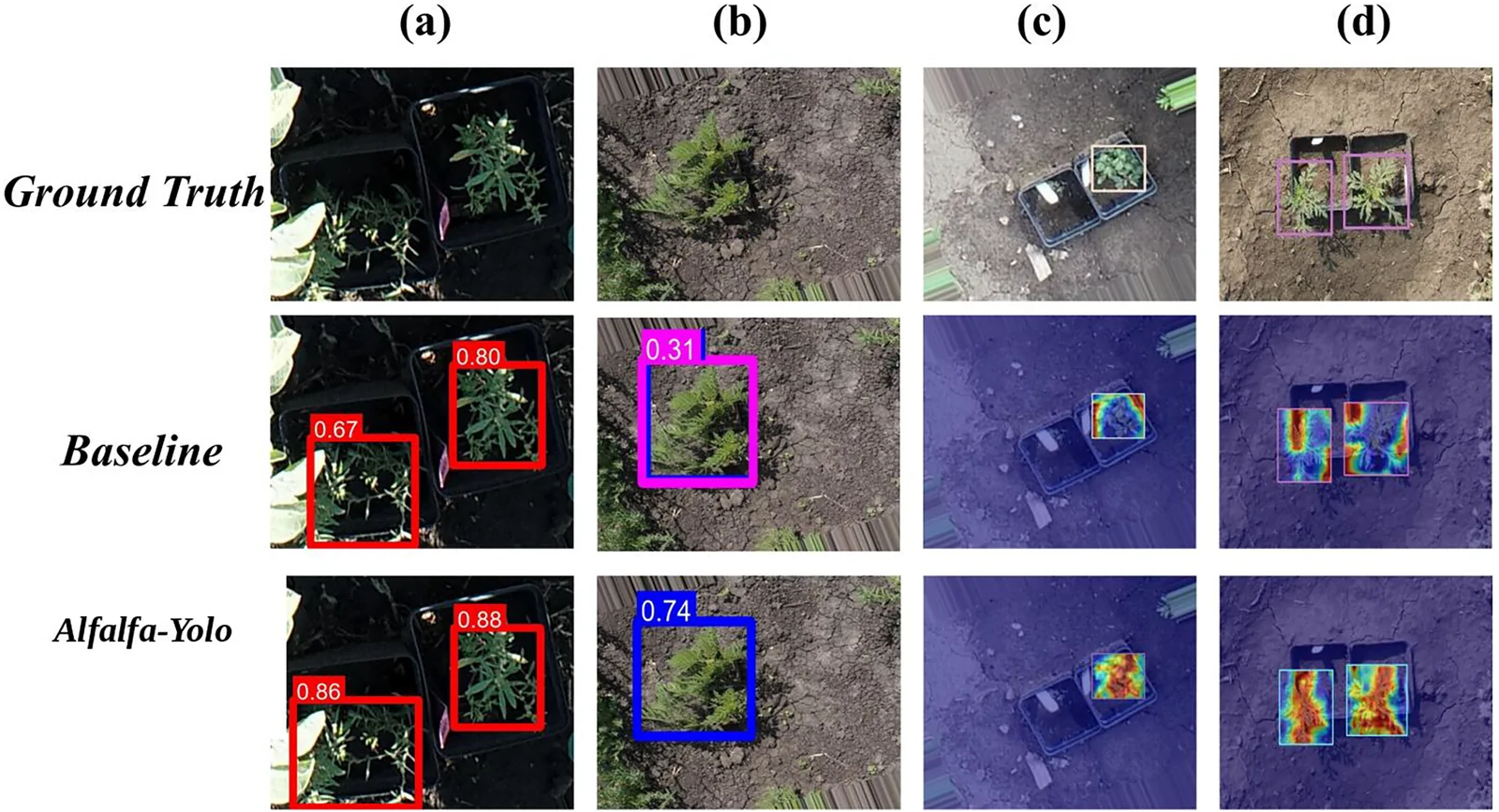
Comparison of the baseline and Alfalfa-Yolo on representative ImageWeeds samples. Columns **(a, b)** present bounding-box detection examples, whereas columns **(c, d)** present Grad-CAM response examples. Warmer colors indicate relatively stronger model responses and cooler colors indicate weaker responses within each image.

In the displayed cases, Alfalfa-Yolo produces more spatially concentrated responses around several weed regions than the Baseline, while the corresponding detection examples show changes in confidence and box placement. These activation maps are interpreted only as qualitative illustrations of feature-response differences. They are not used as quantitative proof of boundary accuracy or cross-domain generalization. The cross-dataset conclusions are instead based on the independent-dataset results in **Table 10** and the strict zero-shot transfer results in **Table 11**.

### 4.7 Deployment experiments

#### 4.7.1 Deployment performance

Deployment efficiency and detection accuracy were evaluated on three computing platforms: NVIDIA RTX 4090, NVIDIA Orin NX, and RK3588. FP32, FP16, and INT8 configurations were tested where supported; because the RK3588 NPU does not support FP32 inference, only FP16 and INT8 were evaluated on that platform. Results are presented in **Table 12** and **Figure 16**.

**Table 12:** Deployment performance of Alfalfa-Yolo on different hardware platforms.

Model	Platform	Quantization	FPS	Latency (ms)	mAP@0.5 (%)	mAP@0.5:0.95 (%)
YOLOv11n (Baseline)	Nvidia RTX 4090	FP32	210.6	4.76	74.4	53.9
		FP32	29.6	33.78	74.4	53.9
	Nvidia Orin NX	FP16	34.7	28.82	73.8	53.4
		INT8	45.8	21.83	73.3	53.1
	RK3588	FP16	15.4	64.94	73.7	53.2
		INT8	23.5	42.55	73.2	53.0
Alfalfa-Yolo (Ours)	Nvidia RTX 4090	FP32	178.7	5.60	77.6	55.9
		FP32	24.3	41.15	77.6	55.9
	Nvidia Orin NX	FP16	30.6	32.68	77.2	55.4
		INT8	38.7	25.84	76.7	53.8
	RK3588	FP16	12.3	81.30	77.3	55.6
		INT8	20.7	48.31	76.9	53.9

**Figure 16:**
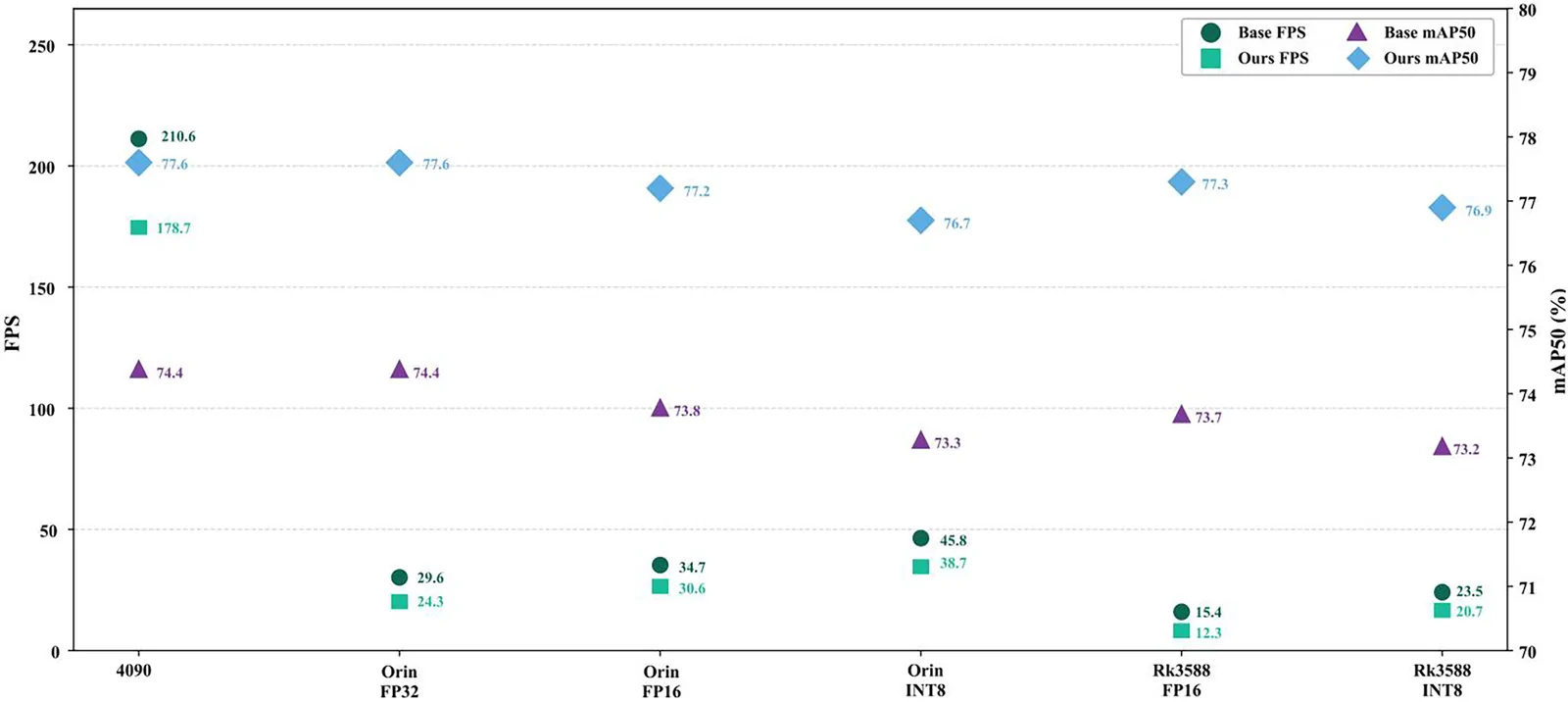
Deployment experiment performance results across different hardware platforms and quantization configurations.

The experimental results show different characteristics for these three hardware platforms. 178.7 FPS inference speed and ultra-low latency of 5.6ms without quantization are achieved by the Nvidia RTX 4090 high-performance computing platform with mAP@0.5 of 77.6% and mAP@0.5:0.95 of 55.9%. The Nvidia Orin NX embedded platform achieves 38.7 FPS under INT8 quantization, with an inference latency of 25.84ms with mAP@0.5 of 76.7%, and accuracy loss of 0.9 percentage points. RK3588 edge computing platform achieves mAP@0.5 of 77.3% under FP16, and after INT8 quantization, FPS improves to 20.7 with latency reduced to 48.31ms and mAP@0.5:0.95 decrease of 1.7 percentage points to 53.9%.

#### 4.7.2 Deployment visualization

Cross-platform detection behavior was further visualized using the same representative alfalfa-field samples on NVIDIA RTX 4090, NVIDIA Orin NX, and RK3588 under their supported precision modes. The resulting detection outputs are shown in **Figure 17**.

**Figure 17:**
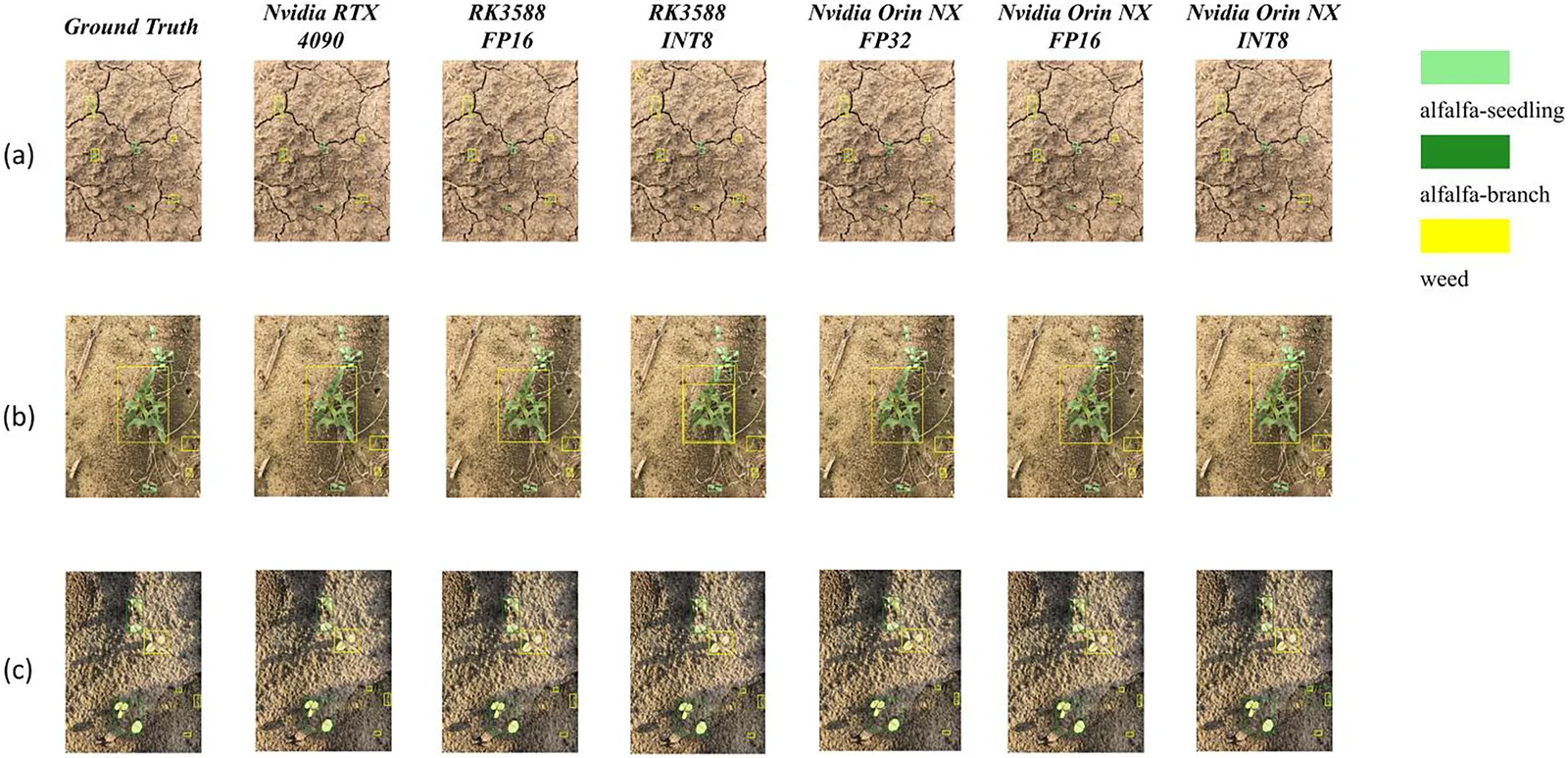
Deployment visualization results across hardware platforms and quantization configurations. Columns from left to right show Ground Truth, NVIDIA RTX 4090, RK3588 FP16, RK3588 INT8, NVIDIA Orin NX FP32, NVIDIA Orin NX FP16, and NVIDIA Orin NX INT8. Rows **(a–c)** correspond to three representative field samples.

The visualization confirms that the model remains operational across the evaluated hardware and quantization settings, although the accuracy-efficiency trade-off differs by platform. RTX 4090 produces outputs close to the reference detections at the highest throughput. On Orin NX, FP16 retains comparatively stable boxes and confidence while providing a useful embedded-device speed, whereas INT8 shows a larger accuracy reduction on some small targets. RK3588 provides lower throughput, and its INT8 configuration exhibits more visible localization degradation in difficult examples. These observations are consistent with the quantitative results in **Table 12** and indicate that the preferred precision setting should be selected according to the accuracy and latency requirements of the target deployment.

## 5 Conclusion

Alfalfa-Yolo addresses attenuation of edge-related detail, insufficient adaptive cross-scale fusion, and spatial-detail loss during upsampling in alfalfa-field weed detection. EPGTNet explicitly preserves and transmits Sobel-derived high-frequency cues within the bounding-box detection framework, MSAFPN adaptively integrates multi-scale representations, and ECSM enhances local spatial information after fixed upsampling through depthwise convolution, channel shuffle, and spatial shift. The boundary-aware description therefore refers to the use of edge-related gradient information rather than explicit pixel-level contour prediction.

On the self-constructed dataset, Alfalfa-Yolo achieves 77.6% mAP@0.5 and 55.9% mAP@0.5:0.95 with 2.67 M parameters. Relative to YOLOv11n, the higher detection accuracy is accompanied by an increase from 6.4 to 8.1 GFLOPs and a throughput decrease from 210.63 to 178.68 FPS, so the model is characterized as parameter-compact rather than uniformly lighter than the baseline. Independent-dataset benchmarking on CottonWeedDet12 and ImageWeeds demonstrates cross-dataset applicability after target-dataset retraining, while the separate zero-shot protocol provides direct evidence of source-only cross-domain transfer behavior. Deployment experiments on RTX 4090, Orin NX, and RK3588 further show that quantization and hardware choice introduce practical accuracy-latency trade-offs.

From an agricultural application perspective, the detector is intended for timely localization of unwanted vegetation to support site-specific weed control. Because all weed species are mapped to a single generic weed class, the current system does not support species-specific herbicide decisions or evaluate discrimination among morphologically similar weed species. Future work will therefore investigate species-level annotation and multi-class weed recognition, together with lightweight Transformer architectures, knowledge distillation, network pruning, and open-world vision-language perception to improve generalization while controlling deployment cost.

## Data Availability

The datasets used and analysed during the current study are available from the corresponding author on reasonable request.

## References

[B1] Carion N. Massa F. Synnaeve G. Usunier N. Kirillov A. Zagoruyko S. (2020). “ End-to-end object detection with transformers”, in: Computer Vision – ECCV 2020. (Cham: Springer International Publishing), 213–229.

[B2] Chen J. Kao S. He H. Zhuo W. Wen S. Lee C.-H. . (2023). “ Run, don’t walk: chasing higher flops for faster neural networks”, in: Proceedings of the IEEE/CVF Conference on Computer Vision and Pattern Recognition. (Los Alamitos, CA: IEEE Computer Society), 12021–12031.

[B3] Chen J. Wang H. Zhang H. Luo T. Wei D. Long T. . (2022). Weed detection in sesame fields using a yolo model with an enhanced attention mechanism and feature fusion. Comput. Electron. Agric. 202, 107412. doi: 10.1016/j.compag.2022.10741242574925

[B4] Dang F. Chen D. Lu Y. Li Z. (2023). Yoloweeds: a novel benchmark of yolo object detectors for multiclass weed detection in cotton production systems. Comput. Electron. Agric. 205, 107655. doi: 10.1016/j.compag.2023.10765542574925

[B5] Deng B. Lu Y. (2026). Toward generalizable crop-weed detection with vision-language models. Smart Agric. Technol. 15, 102512. doi: 10.1016/j.atech.2026.10251242574925

[B6] Feng Y. Huang J. Du S. Ying S. Yong J.-H. Li Y. . (2024). Hyper-yolo: when visual object detection meets hypergraph computation. IEEE Trans. Pattern Anal. Mach. Intell. 47, 2388–2401. doi: 10.1109/TPAMI.2024.3524377 40030788

[B7] Jia Z. Zhang M. Yuan C. Liu Q. Liu H. Qiu X. . (2024). Adl-yolov8: a field crop weed detection model based on improved yolov8. Agronomy 14, 2355. doi: 10.3390/agronomy1410235530654563

[B8] Jocher G. . (2020). Yolov5: a fresh state-of-the-art implementation. Available online at: https://github.com/ultralytics/yolov5 (Accessed October 7, 2026).

[B9] Jocher G. . (2023). Yolov8: a fresh state-of-the-art implementation. Available online at: https://github.com/ultralytics/ultralytics (Accessed October 7, 2026).

[B10] Khanam R. Hussain M. (2024). Yolov11: an overview of the key architectural enhancements. arXiv preprint arXiv:2410.17725. doi: 10.48550/arXiv.2410.17725

[B11] Lee S. Choi J. Kim H. J. (2025). “ Efficientvim: efficient vision mamba with hidden state mixer based state space duality”, in: Proceedings of the Computer Vision and Pattern Recognition Conference. (Los Alamitos, CA: IEEE Computer Society), 14923–14933.

[B12] Lei M. Li S. Wu Y. Hu H. Zhou Y. Zheng X. . (2025). Yolov13: real-time object detection with hypergraph-enhanced adaptive visual perception. arXiv [preprint] arXiv:2506.17733

[B13] Li C. Li L. Jiang H. Weng K. Geng Y. Li L. . (2022). Yolov6: a single-stage object detection framework for industrial applications. arXiv [preprint]. arXiv:2209.02976

[B14] Li D. Li L. Chen Z. Li J. (2024). Shift-convnets: small convolutional kernel with large kernel effects. arxiv. arXiv preprint arXiv:2401.12736 (2024). doi: 10.1145/3664647.3681088

[B15] Li H. (2024). “ Rethinking features-fused-pyramid-neck for object detection”, in: Computer Vision – ECCV 2024. (Cham: Springer Nature Switzerland), 74–90.

[B16] Li W. Guo H. Liu X. Liang K. Hu J. Ma Z. . (2024). “ Efficient face super-resolution via wavelet-based feature enhancement network”, in: Proceedings of the 32nd ACM International Conference on Multimedia. (New York, NY: ACM), 4515–4523.

[B17] Liakos K. G. Busato P. Moshou D. Pearson S. Bochtis D. (2018). Machine learning in agriculture: a review. Sensors 18, 2674. doi: 10.3390/s18082674 30110960 PMC6111295

[B18] Lin T.-Y. Dollár P. Girshick R. He K. Hariharan B. Belongie S. (2017). “ Feature pyramid networks for object detection”, in: Proceedings of the IEEE Conference on Computer Vision and Pattern Recognition. (Los Alamitos, CA: IEEE Computer Society), 2117–2125.

[B19] Liu W. Anguelov D. Erhan D. Szegedy C. Reed S. Fu C.-Y. . (2016). “ Ssd: single shot multibox detector”, in: Computer Vision – ECCV 2016. (Cham: Springer International Publishing), 21–37.

[B20] Lu W. Chen S.-B. Li H.-D. Shu Q.-L. Ding C. H. Tang J. . (2025). “ Legnet: a lightweight edge-gaussian network for low-quality remote sensing image object detection”, in: Proceedings of the IEEE/CVF International Conference on Computer Vision. (Los Alamitos, CA: IEEE Computer Society), 2844–2853.

[B21] Ma C. Chi G. Ju X. Zhang J. Yan C. (2025). Yolo-cwd: a novel model for crop and weed detection based on improved yolov8. Crop Prot. 192, 107169. doi: 10.1016/j.cropro.2025.10716942574925

[B22] Moyer J. R. Schaalje G. B. Bergen P. (1991). Alfalfa (medicago sativa) seed yield loss due to canada thistle (cirsium arvense). Weed Technol. 5, 723–728. doi: 10.1017/s0890037x0003375341292463

[B23] Murad N. Y. Mahmood T. Forkan A. R. M. Morshed A. Jayaraman P. P. Siddiqui M. S. (2023). Weed detection using deep learning: a systematic literature review. Sensors 23, 3670. doi: 10.3390/s23073670 37050730 PMC10098587

[B24] Nasir M. F. Rehman M. U. Hussain I. (2026). Vision-language models for zero-shot weed detection and visual reasoning in UAV-based precision agriculture. Front. Plant Sci. 16, 1735096. doi: 10.3389/fpls.2025.1735096 41695537 PMC12894358

[B25] Olsen A. Konovalov D. A. Philippa B. Ridd P. Wood J. C. Johns J. . (2019). Deepweeds: a multiclass weed species image dataset for deep learning. Sci. Rep. 9, 2058. doi: 10.1038/s41598-018-38343-3 30765729 PMC6375952

[B26] Peng G. Wang K. Ma J. Cui B. Wang D. (2025). Agri-yolo: a lightweight model for corn weed detection with enhanced yolo v11n. Agriculture 15, 1971. doi: 10.3390/agriculture1518197130654563

[B27] Rai N. Mahecha M. V. Christensen A. Quanbeck J. Zhang Y. Howatt K. . (2023). Multi-format open-source weed image dataset for real-time weed identification in precision agriculture. Data Brief 51, 109691. doi: 10.1016/j.dib.2023.109691 37920388 PMC10618417

[B28] Redmon J. Divvala S. Girshick R. Farhadi A. (2016). “ You only look once: unified, real-time object detection”, in: Proceedings of the IEEE Conference on Computer Vision and Pattern Recognition. (Los Alamitos, CA: IEEE Computer Society), 779–788.

[B29] Ren S. He K. Girshick R. Sun J. (2015). Faster r-cnn: towards real-time object detection with region proposal networks. Adv. Neural Inf. Process. Syst. 28, 91–99. 10.1109/TPAMI.2016.2577031 27295650

[B30] Sharma A. Kumar V. Longchamps L. (2024). Comparative performance of yolov8, yolov9, yolov10, yolov11 and faster r-cnn models for detection of multiple weed species. Smart Agric. Technol. 9, 100648. doi: 10.1016/j.atech.2024.10064842574925

[B31] Shi Z. Hu J. Ren J. Ye H. Yuan X. Ouyang Y. . (2025). “ Hs-fpn: high frequency and spatial perception fpn for tiny object detection”, in: Proceedings of the AAAI Conference on Artificial Intelligence. (Washington, DC: AAAI Press), vol. 39, 6896–6904.

[B32] Shorten C. Khoshgoftaar T. M. (2019). A survey on image data augmentation for deep learning. J. Big Data 6, 1–48. doi: 10.1186/s40537-019-0197-0 34306963 PMC8287113

[B33] Tian Y. Ye Q. Doermann D. (2025). Yolov12: attention-centric real-time object detectors. arXiv [preprint]. arXiv:2502.12524 (2025)

[B34] Veeranampalayam Sivakumar A. N. Li J. Scott S. Psota E. Jhala A. J. Luck J. D. . (2020). Comparison of object detection and patch-based classification deep learning models on mid-to late-season weed detection in uav imagery. Remote Sens. 12, 2136. doi: 10.3390/rs1213213630654563

[B35] Wang A. Chen H. Liu L. Chen K. Lin Z. Han J. . (2024). Yolov10: real-time end-to-end object detection. arXiv [preprint] arXiv:2405.14458

[B36] Wang A. Zhang W. Wei X. (2019). A review on weed detection using ground-based machine vision and image processing techniques. Comput. Electron. Agric. 158, 226–240. doi: 10.1016/j.compag.2019.02.00542574925

[B37] Wang J. Qi Z. Wang Y. Liu Y. (2025). A lightweight weed detection model for cotton fields based on an improved yolov8n. Sci. Rep. 15, 457. doi: 10.1038/s41598-024-84748-8 39747358 PMC11696358

[B38] Wu Z. Chen Y. Zhao B. Kang X. Ding Y. (2021). Review of weed detection methods based on computer vision. Sensors 21, 3647. doi: 10.3390/s21113647 34073867 PMC8197187

[B39] Xu W. Zheng S. Wang C. Zhang Z. Ren C. Xu R. . (2025). Samamba: adaptive state space modeling with hierarchical vision for infrared small target detection. Inf. Fusion 124, 103338. doi: 10.1016/j.inffus.2025.10333842574925

[B40] Yang Z. Khan Z. Shen Y. Liu H. (2025). Gtdr-yolov12: optimizing yolo for efficient and accurate weed detection in agriculture. Agronomy 15, 1824. doi: 10.3390/agronomy1508182430654563

[B41] Yang J. Wang Y. Chen Y. Yu J. (2022). Detection of weeds growing in alfalfa using convolutional neural networks. Agronomy 12, 1459. doi: 10.3390/agronomy1206145930654563

[B42] Zhang Y. Wang L. (2025). Advances in basic biology of alfalfa (medicago sativa l.): a comprehensive overview. Hortic. Res. 12, uhaf081. doi: 10.1093/hr/uhaf081 40343348 PMC12058308

[B43] Zhao Y. Lv W. Xu S. Wei J. Wang G. Dang Q. . (2024). “ Detrs beat yolos on real-time object detection”, in: Proceedings of the IEEE/CVF Conference on Computer Vision and Pattern Recognition. (Los Alamitos, CA: IEEE Computer Society), 16965–16974.

