# Supplementary material for: Alfalfa-Yolo: a boundary-aware lightweight framework for real-time weed detection in alfalfa fields
Source: Front Plant Sci. 2026 Oct 15;17:1955970. doi: 10.3389/fpls.2026.1955970 (PMC13716862; doi:10.3389/fpls.2026.1955970)
Supplement: Supplementary file 1 [file DataSheet1.pdf]

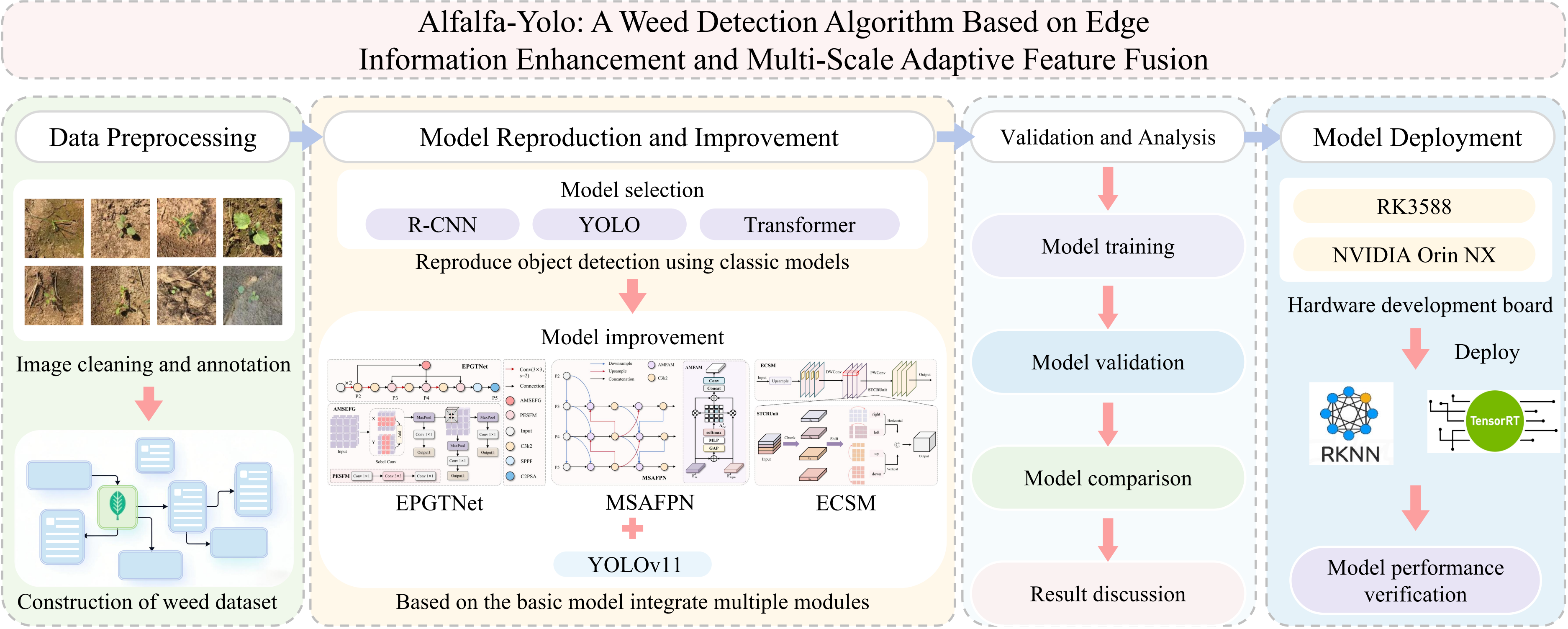

Alfalfa-Yolo: A Weed Detection Algorithm Based on Edge Information Enhancement and Multi-Scale Adaptive Feature Fusion
Data Preprocessing
Image cleaning and annotation
Construction of weed dataset
Model Reproduction and Improvement
Model selection
R-CNN
YOLO
Transformer
Reproduce object detection using classic models
Model improvement
EPGTNet
MSAFPN
ECSM
YOLOv11
Based on the basic model integrate multiple modules
Validation and Analysis
Model training
Model validation
Model comparison
Result discussion
Model Deployment
RK3588
NVIDIA Orin NX
Hardware development board
Deploy
RKNN
TensorRT
Model performance verification
